# Time- and Temperature-Resolved Triplet Dynamics in Tungsten Iodide Clusters

**DOI:** 10.1021/acs.inorgchem.5c02344

**Published:** 2025-10-06

**Authors:** Philipp Frech, Wolfgang Leis, Florian Pachel, Michael Seitz, H.-Jürgen Meyer, Marcus Scheele

**Affiliations:** † Institute of Physical and Theoretical Chemistry, 9188 University of Tübingen, Auf der Morgenstelle 18, 72076 Tübingen, Germany; ‡ Institute of Inorganic Chemistry, 9188 University of Tübingen, Auf der Morgenstelle 18, 72076 Tübingen, Germany; § Section for Solid State and Theoretical Inorganic Chemistry, Institute of Inorganic Chemistry, 9188 University of Tübingen, Auf der Morgenstelle 18, 72076 Tübingen, Germany

## Abstract

This study comprehensively investigates the excited-state dynamics of two tungsten iodide prototype clusters, [(W_6_I_8_)­I_6_]^2–^ and [(W_6_I_8_)­(TFA)_6_]^2–^ (TFA = trifluoroacetate), utilizing a combination of ultrafast transient absorption spectroscopy from 200 fs up to 400 μs and temperature-dependent emission spectroscopy from 4 to 340 K. Both clusters exhibit rapid intersystem crossing occurring within 6 ps, populating triplet states that subsequently deactivate through emission or dynamical bimolecular quenching involving molecular oxygen. The temperature-dependent emission behavior aligns well with a group-theoretical spin-sublevel model, indicating three distinct emissive sublevels. However, contrary to previous findings in molybdenum-based clusters, no additional splitting of the lowest triplet states was observed experimentally. Time-dependent density functional theory calculations highlight substantial excited-state geometrical distortions, suggesting limitations in sole group-theoretical descriptions. Instead, we propose a relativistic model with three thermally accessible excited-state geometries, each presenting three triplet sublevels.

## Graphical abstract

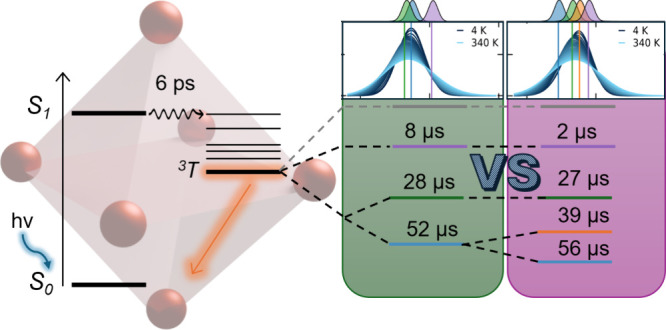

## Introduction

Octahedral transition metal halide clusters have long been of interest due to their intriguing structural diversity and remarkable optical properties. As illustrated in Chart 1, they are characterized by a central metal octahedron (M = Mo­(II), W­(II), Re­(III)) surrounded by eight tightly bound face-capping inner ligands (X = halogenide for M­(II) or chalcogenide for M­(III)) and six exchangeable *apical* outer ligands (L = X, RCOO^–^, tosyl^–^, ···) as [(M_6_X_8_)­L_6_]^2–^.^1–3^ In 1981, the excitation of (TBA)_2_[(Mo_6_Cl_8_)­Cl_6_] (TBA = *tetra*-*n*-butylammonium) with blue to UV light was demonstrated to yield a bright and long-lived luminescence in the far-red to NIR.^4^ Upon exposure to oxygen, the excited state can be efficiently quenched through the generation of singlet oxygen, which was first described in 1990.^5^ In the following years, various ligand-substituted molybdenum and rhenium-based clusters have been synthesized and optimized toward higher luminescence quantum yields, enhancing their potential for oxygen sensing, bioimaging, and cancer therapy.^2,6–16^ Since 2016, corresponding tungsten iodide clusters have been reported with photoluminescence and singlet oxygen generation quantum yields compared to molybdenum-based counterparts.^17–21^

**Chart 1:**
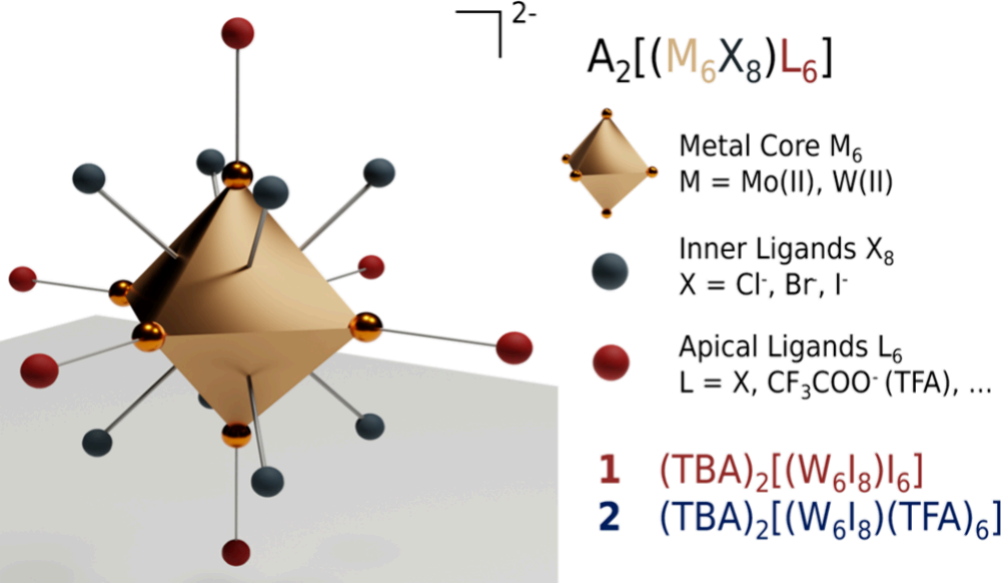
Structure of Octahedral Hexanuclear M­(II) Clusters of the Type [(M_6_X_8_)­L_6_]^2–^

The origin of the unusual broad emission and high sensitivity to temperature and ligand has been discussed by several research groups. There is evidence that these clusters undergo intersystem crossing (ISC) into emissive triplet states, which can transfer their energy to molecular oxygen under the formation of singlet oxygen.^5,17,19,22–24^ However, studies regarding the excited singlet state are limited. Time-resolved photoluminescence spectroscopy on crystalline (TBA)_2_[(Mo_6_Cl_8_)­Cl_6_] indicates singlet-state emission between 600 and 1100 ps.^25^ Contrarily, transient absorption spectroscopy (TAS) of molybdenum clusters in solution implied an ISC within 2 ps, followed by a hot triplet cooling within 5 ps.^24^ For the cluster cation [(W_6_I_8_)­(CH_3_CN)_6_]^4+^, we recently reported ISC times of 2 ps, followed by an additional relaxation within 90 ps.^26^

Most importantly, the nature of the emissive triplet states has been debated controversially, with input from several theoretical and experimental investigations. Azumi and Saito discussed the splitting of several low-energy states due to electron repulsion and spin–orbit coupling (SOC).^22^ In their nonrelativistic calculations, they identified the ^3^T_1u_ state as the emissive state, splitting into four sublevels T_2u_, E_u_, T_1u_, and A_1u_ in O_h_ geometry (compare Chart 2, green patch). Due to an allowed dipole transition, T_1u_ is predicted to have the strongest emission, outcompeting the higher lying forbidden A_1u_ state. This model, using three emissive states, was able to model the temperature-dependent emission, e.g., for Mo­(II),^27^ Ru­(II),^28^ Pt­(II),^29^ and Ir­(II) complexes.^30^ However, the need for a fourth emissive component at temperatures below 10 K emerged recently for Mo­(II)-based clusters.^31^ Kitamura and co-workers studied a comprehensive [(Mo_6_X_8_)­L_6_]^2–^ cluster series and found that four emissive sublevels not only modeled the data better but also revealed strong correlations between the ligand nature and the strength of the cluster sublevel splitting.^32–34^ They rationalized this fourth component by symmetry lowering due to Jahn–Teller distortion from O_h_ to D_4h_, resulting in a splitting of the T_2u_ state into B_2u_ and E_u_ (compare Chart 2, purple patch). Following their notation, the model using three emissive sublevels due to SOC will be named the φ_n_ model, while the ϕ_*n*_ model uses four emissive sublevels due to a combination of SOC and symmetry lowering. However, for [(Re_6_S_8_)­Cl_6_]^4–^ and [(W_6_Cl_8_)­Cl_6_]^2–^, the authors could not identify a statistical justification of the ϕ_*n*_ model over the φ_n_ model.^31^ Relativistic quantum chemical density functional theory (DFT) calculations support symmetry lowering of the excited triplet states, leading to a loss of degeneracy from the O_h_ symmetry initially assumed by the φ_n_ model. Besides the symmetry reduction to D_4h_, further distortions to D_2h_, C_2v_, or C_s_ are also observed for Mo­(II), W­(II), and Re­(III) clusters, which are not accounted for by the ϕ_*n*_ model (compare blue patch in Chart 2).^3,35–37^ Complementary experimental evidence for such symmetry-breaking relaxations comes from the combined time-resolved photoluminescence and quantum-chemical study of Costuas et al. on [(Mo_6_Br_8_)­Br_6_]^2–^, revealing at least two distinct emissive triplet states arising from sizable geometrical relaxations in the triplet state.^3,38^ Furthermore, they revealed that the emission properties can depend on the excitation wavelength and the irradiation type, further emphasizing that vibronic coupling and SOC alone cannot explain the observed emission properties.

**Chart 2:**
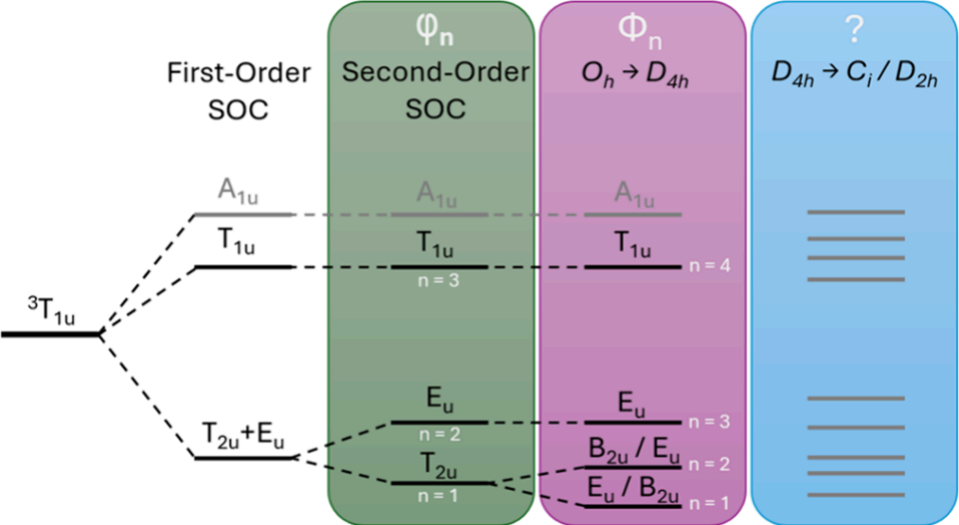
Lowest-Lying Excited Triplet State Spin Sublevel Models of M­(II) Clusters^22,31^

Resolving the nature and splitting of the emissive triplet sublevels is essential as it directly impacts oxygen sensing accuracy, photodynamic therapy efficiency, and the reliability of bioimaging techniques. Clarifying this photophysical model will enable a more precise tuning of cluster properties, significantly enhancing their practical applicability in biomedical contexts.

Although the emission properties of [W_6_X_8_)­L_6_]^2–^ have been extensively studied in the past decade, low-temperature or excited-state studies remain limited. Aside from the mentioned study on crystalline (TBA)_2_[(W_6_Cl_8_)­Cl_6_],^31^ we are not aware of any other experimental research addressing this fundamental question about the nature of the emissive triplet states in W­(II) cluster complexes.

Here, we approach this long-standing question by analyzing the prototype (TBA)_2_[(W_6_I_8_)­I_6_] cluster **1** (red) and (TBA)_2_[(W_6_I_8_)­(TFA)_6_] (TFA = *tri*fluoroacetate) cluster **2** (blue) in solution and dispersed in poly­(methyl methacrylate) (PMMA). We combine ultrafast TAS, nanosecond TAS, and temperature-dependent emission (PL) spectroscopy with scalar relativistic time-dependent DFT (TDDFT) calculations to draw a clearer picture of the excited-state properties of W_6_I_8_ cluster complexes. As a result, we report ISC times, oxygen quenching rates, and triplet splitting characteristics based on the tested models. We find that three sublevels are sufficient to describe the experimental observations of tungsten iodide clusters. However, calculated excited-state geometries indicate a complete loss of degeneracy, resulting in three thermally accessible triplet sets. Each of these nine states contributes to the ensemble emission, albeit through slightly different excited-state geometries. To our knowledge, this is the first spectroscopic quantification of ISC rates and the triplet sublevel splitting in anionic [(W_6_I_8_)­L_6_]^2–^ clusters and its mechanistic correspondence to distinct relaxed excited-state geometries.

## Experimental Section

### Sample Preparation and Containers

**1** and **2** were synthesized following published protocols.^17,39^ Briefly, WCl_6_ and SiI_4_ react at 120 °C, forming W_3_I_12_, which can be converted with CsI at 550 °C to Cs_2_[(W_6_I_8_)­I_6_]. The cation can be exchanged in solution with tetra-*n*-butylammonium iodide ((TBA)­I), forming **1**, and the *apical* ligand can be exchanged via salt metathesis with Ag­(TFA), forming **2**.

All spectroscopic measurements (steady-state absorption and emission, emission lifetime, and TA) were conducted on samples prepared either in acetonitrile solution or embedded in PMMA thin films, as described below.

For the spectroscopic measurements in solution, HPLC-grade acetonitrile was distilled over molecular sieves and subsequently purified by neutral Al_2_O_3_ (Brockmann activity I) chromatography in a glovebox. The cluster concentrations for the TA and steady-state absorbance experiments were set to 60 μM (optical density at 350 nm (OD_350_) ≅ 0.1) and 30 μM for the PL experiments, respectively.

For the spectroscopic measurements in a PMMA thin film, the microcrystalline cluster and PMMA were dissolved in absolute acetone and mixed to achieve a cluster concentration of 0.33 mg/mL or 100 μM and a PMMA concentration of 8 wt %. The solution was spin-coated at 20 rpm on a glass substrate and dried under vacuum for 12 h (see Figure S1.1).

Steady-state absorption and emission experiments at room temperature were performed in acetonitrile solution and in a PMMA thin film.

The TA experiments were performed in 2 mm quartz glass cuvettes from Helma Analytics, with a home-built opening featuring a stopcock with a polytetrafluoroethylene key and a septum (see Figure S1.2). This way, the oxygen levels can be adjusted by gently bubbling a premixed N_2_/O_2_ stream from Westfalen Gas (5.0 purity) through a needle for 30 min.

Regular borosilicate glass NMR tubes with a screw cap and septum were used for the room-temperature and 77 K PL experiments. The oxygen level was adjusted in the same way as that for the TA experiments.

For the variable-temperature PL measurements in acetonitrile, the cluster solution was transferred into a borosilicate glass NMR tube with J. Young valve, and gases were removed through five freeze–pump–thaw cycles. While the solution was frozen in liquid nitrogen, the upper section of the tube was melted off by using a gas burner.

### Spectroscopic Measurements

Steady-state absorption measurements were performed on a UV–vis–NIR Cary 5000 spectrometer from Agilent Technologies.

### Emission Spectroscopy

Steady-state spectra were measured on a Horiba Fluorolog-3 spectrofluorimeter equipped with a 450 W xenon lamp for steady-state measurements. For fluorescence lifetime measurements, a pulsed laser (PicoQuant LDH-P-C-375, λ_exc_ = 371 ± 2 nm, pulse width ca. 50 ps FWHM, *P*_max,avg_ ≈ 5 mW) was used. For phosphorescence lifetime measurements, excitation was performed with a pulsed xenon lamp (pulse width ca. 2 μs FWHM) using the excitation monochromator pathway or using adapted burst pulse sequences (PicoQuant PDL820) with the given laser setup. Emitted light was detected by a Hamamatsu R13456 PMT (UV/vis/NIR, 200 nm < λ_em_ < 950 nm). A double grating monochromator 320DFX (1200 grooves/nm, blazed at 330 nm or 600 grooves/nm, blazed at 750 nm) was used for spectral selection in the excitation path, while in the visible emission path, the single grating monochromators iHR550 (1200 grooves/mm, blazed at 500 nm; 950 grooves/mm, blazed at 900 nm; 1800 grooves/mm, blazed at 500 nm) and iHR320 (600 grooves/mm, blazed at 1000 nm) were used. To avoid higher-order excitation light, long-pass filter glass plates (Schott, 3 mm thickness) were used in the emission beam path when needed. To avoid longer-wavelength emission of LDH-P-C-375, we used a 475 nm Techspec (Edmund optics) short-pass filter (OD > 4.0) in front of the laser diode.

To obtain more stable lifetime results for the variable-temperature experiments, the determination of the PL lifetimes was realized as a TRES (time-resolved emission spectra) measurement. This involved the determination of the luminescence decays at 5–6 wavelengths of the emission band and the global fitting of these decays.

### Cryogenic Emission Spectroscopy

For 77 K PL spectra, solvents forming a glass upon rapid cooling were preferred but were not compulsively used. For cooling and spectrum detection, the sample tube within a quartz dewar (Horiba/PTI) filled with liquid nitrogen was placed into the light path.

Temperature-dependent PL measurements in the range of 4–340 K were carried out using a Horiba Fluorolog-3 with the same general instrument configuration as described above but equipped with a closed-cycle helium cryostat CS204-X1.Al operated with a helium compressor module ARS-4HW (Advanced Research Systems) and a temperature controller LS-335 (Lake Shore Cryotronics). The cryostat was configured with high-purity quartz windows (W-X1-HPQ) and with a custom-made copper sample holder for borosilicate glass tubes (outer diameter 5 mm, length ca. 2.5 cm). The cryostat volume was evacuated with a regular oil pump, reaching at least 10^–2^ hPa during the operation. The temperature was measured by using the standard silicon diode sensors of the cryostat attached to the base of the sample holder. The sample tubes containing the samples were about 5 cm below the temperature sensor.

### Transient Absorption Measurements

TAS was conducted with a fs TA spectrometer HELIOS Fire and the ns-TA spectrometer EOS Fire, both from Ultrafast Systems. 90 fs laser pulses at a repetition rate of 1 kHz and 800 nm central wavelength were generated with an Astrella-F ultrafast Ti/sapphire amplifier from Coherent. The fundamental beam was split into a pump and probe beam, and the wavelength of the pump was adjusted to 350 nm by using the fourth harmonic of the signal in the optical parametric amplifier Apollo-T from Ultrafast Systems. The pump intensity was set between 0.5 and 1 μJ/pulse to exclude nonlinear dynamics (photons per molecule < 2%). Impure excitation light and scattered light were purged with appropriate filters. For the fs experiments, a white-light continuum (WLC) from 320 to 650 nm was generated with a CaF_2_ crystal, while for the ns experiments, the WLC was generated with a photonic crystal fiber. The delay between the pump and probe was altered by a mechanical delay stage for the ultrafast experiments up to 2 ns, while for the ns pump–probe measurements, the delay up to 450 μs was controlled electronically. To account for energy fluctuations, a reference channel for the probe was used, and multiple scans were acquired and averaged. The samples were stirred, and the integrity of the samples was confirmed with absorption measurements before and after the experiments.

### TDDFT Calculations

DFT and TDDFT calculations were performed in ORCA 6.^40^ For the calculations, the hybrid functional PBE0 was employed alongside Grimme’s D4 dispersion correction, and scalar relativistic effects were accounted for using the eXact Two-Component (X2C) Hamiltonian.^41–53^ Specifically, excited-state geometry optimizations used the diagonal local approximation to the unitary transformation matrix (DLU approximation) within the X2C calculation.^54^ A triple-ζ basis set optimized for X2C (X2C-TZVPPALL) and its corresponding auxiliary set (X2C/J) were used to ensure accurate representation of the electronic structure.^55,56^ Spin–orbit effects were handled with the RI-SOMF­(1X) approach, and a Gaussian finite nucleus model was applied.^57^ The strict self-consistent field (SCF) convergence criterion, TightSCF, was used alongside TightOPT for geometry optimization.

As initial input geometry, a structure from single-crystal X-ray diffraction was used. The preoptimized structure DFT (PBE0|X2C-TZVPPALL) was used for the TDDFT geometry optimizations of the structures of ground and excited states, including spin–orbit coupling. For TDDFT calculations of the spectra, typically, the 50 lowest singlet and triplet excited states/transitions (50 roots) were requested, corresponding to 200 transitions in the spin–orbit-coupled TDDFT calculation.

### Model Fitting and Software

Emission data were acquired using FluorEssence and DataStation, both from HORIBA Scientific. PL lifetime data analysis (deconvolution, statistical parameters, etc.) was performed using the DAS6 software package from Horiba. The decay data were analyzed by tail-fitting (exclusion of the lamp-pulse region). The emission lifetime was calculated by globally fitting the emission kinetics at least at five different wavelengths using DAS6 from HORIBA Scientific.

TA data were acquired with the software Surface Xplorer 4.5, while the data were processed and fitted in Python 3.11 using the packages NumPy, SciPy, Matplotlib, and lmfit.^58^

Sublevel-model fitting of the temperature-dependent emission data was performed in Python using lmfit, and the posterior distribution of parameters was evaluated using emcee.^59^

## Results and Discussion

### Steady-State Properties

Before analyzing the excited-state properties in acetonitrile and PMMA, **1** and **2** were examined by UV/vis absorption and photoluminescence spectroscopy to manifest a possible influence of the solvent and PMMA. This comparison is depicted in Figure 1A. At room temperature, we observe the first broad absorption shoulder at around 417 nm and a second band at 355 nm for both clusters, independent of the matrix. For **1**, the asymmetric emission is centered around 700 nm in acetonitrile and 695 nm in PMMA matrix, respectively, while for **2**, the emission maximum is blue-shifted at 675 nm for acetonitrile and PMMA. The values in solution at room temperature are consistent with other reports.^17,19^ The comparable spectra regardless of the medium lead us to the conclusion that the clusters are well dispersed in the PMMA matrix and that cluster–cluster or cluster–polymer interactions are negligible for the concentrations used in this study. The emission lifetimes at 298 K in solution are in line with the lifetimes in PMMA for both compounds, which further support this claim. The photophysical properties of **1** and **2** are summarized in Table 1.

**Table 1:** Photophysical Properties of **1** and **2** at 297 K in Acetonitrile and PMMA

compound		λ_max_/nm	FWHM/meV	τ_T_ (PL)/μs	τ_T_ (TAS)/μs	*k*_q_ (PL)/10^8^ M^–1^ s^–1^	*k*_q_ (TAS)/10^8^ M^–1^ s^–1^	ISC/ps
1	(TBA)_2_[(W_6_I_8_)I_6_]	700, 695^a^	438, 434^a^	27, 24^a^	22	9.9	9.2	4
2	(TBA)_2_[(W_6_I_8_)(TFA)_6_]	675, 675^a^	481, 447^a^	38, 34^a^	33	3.5	3.7	6

a Corresponding data in PMMA.

**Figure 1:**
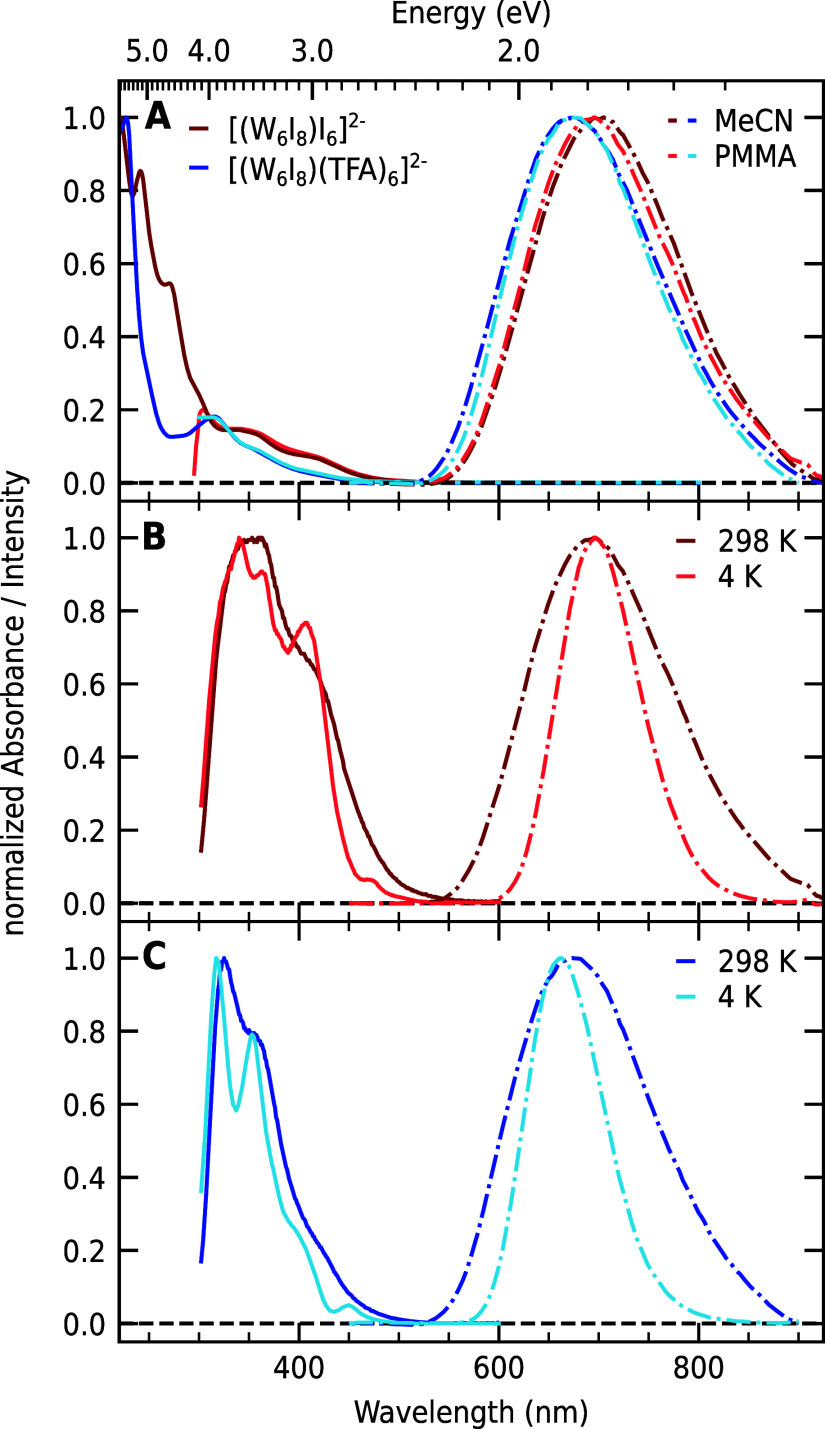
Optical properties of **1** (red) and **2** (blue). (A) Absorbance (straight) and emission (dashed) at 298 K in an acetonitrile (dark) and PMMA matrix (bright). The excitation wavelength was 370 nm for all of the emission measurements. Note that the glass substrate used for the PMMA films has a wavelength cutoff of 310 nm. (B,C) Excitation (straight) and emission (dashed) spectra of **1** and **2** in PMMA at room temperature (dark) and at 4 K (bright).

Figure 1B,C compares the excitation and emission spectra of **1** and **2** at 4 K and room temperature. We note that the excitation spectra at room temperature in PMMA match well the absorbance spectra from Figure 1A. At 4 K, the peaks get narrower, revealing maxima at 470, 408, and 363 nm for **1** and 450, 400, and 353 nm for **2**.

Likewise, the emission bands get narrower and more symmetrical at 4 K, accompanied by a hypsochromic shift of 13 nm for 2. The temperature dependence is consistent with published emission data on related compounds^26,31–34^ and will be analyzed in detail below.

### Ultrafast Spectroscopy of Excited States

We sought to identify the correct sublevel model for **1** and **2**, respectively, by a variety of complementary time-resolved spectroscopy experiments. We begin our investigation with femtosecond TAS, where we follow the excited-state properties and relaxation pathways for both clusters from 100 fs up to 2 ns (Figure 2). Figure 2A shows a background-corrected femtosecond 2D transient absorption spectrum of **1** in acetonitrile. The analysis of the femtosecond TAS experiments of **2** can be found in S2. The differential absorbance is color-coded, where blue means a loss in absorbance of the excited sample compared to the unexcited sample and red represents a gain in absorbance. Upon excitation with 90 fs laser pulses at 350 nm, we find broad excited-state absorption (ESA) over the whole spectral range superimposed with ground-state bleaching at the steady-state absorbance maxima due to Pauli blocking and ground-state depletion (compare the absorbance spectrum in red above the 2D plot). Analysis of the kinetics at 500 nm (Figure 2B) reveals three distinct components, which can be fitted by a sequential exponential model with lifetimes of 130 fs, 4 ps, and a lifetime larger than the measurement window (coefficient of determination *r* ^2^ = 0.99). More details on the selected sequential model and the variability in the fitting results can be found in S2. Global fitting over the whole spectral range with the same model gives comparable lifetimes (*r* ^2^ = 0.98), with the corresponding evolution-associated difference spectra (EADS) shown in Figure 2C. We note that the second and third components have a similar spectral evolution, while the first differs more. The ultrafast first component has a lifetime close to the instrument response function (IRF) of our system of 200 fs and is overlaid by the solvent response and so-called coherent artifacts, which are very prominent within the first 500 fs. The TA spectrum of the pure solvent under the same experimental conditions is analyzed in S2. Since the first component mainly originates from the solvent response and coherent artifacts, we will not include it in the following analysis.

**Figure 2:**
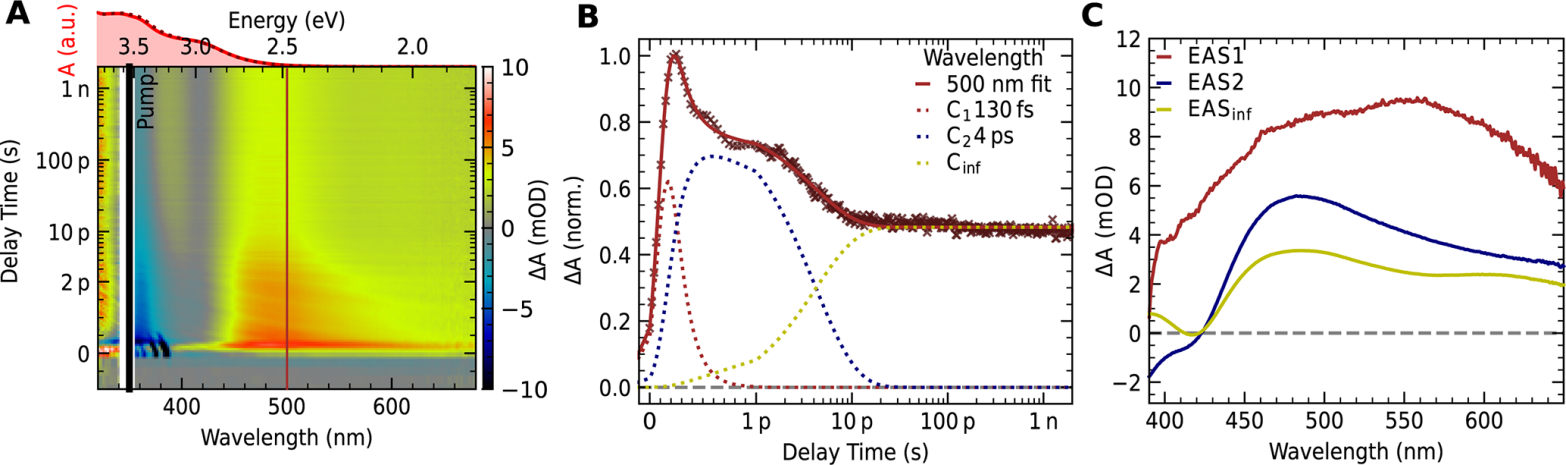
(A) fs TA-hyperspectrum of **1** in acetonitrile after excitation at 350 nm and room temperature. The background is corrected to account for the overlying emission starting around 550 nm. The magnitude and sign of the differential absorption Δ*A* (excited state – ground state) is color-coded on the right-hand side. The delay time between pump and probe beam within the first two ps is displayed with a linear scale, followed by a logarithmic scale until the end of the measurement window of 2 ns. The corresponding steady-state absorption spectrum is shown in red above the 2D plot. (B) Kinetic trace of (A) at 500 nm with the corresponding local exponential fit (straight) and the underlying three sequential exponential functions (dotted). (C) Evolution-associated spectra of the global analysis of panel (A). The first component (red) decays within 150 fs to the second component (blue), which further decays in 4 ps to the third component (yellow), with a lifetime significantly longer than 2 ns.

### Excited Triplet State Properties and Oxygen Quenching

In continuation of our time-resolved analysis, we now use nanosecond TAS to specifically study the long-lived component in further detail. We find that under inert conditions, the third component decays monoexponentially with lifetimes of 22 μs for **1** and 33 μs for **2**. This component can be quenched reversibly by oxygen down to 90 ns for **1** and 250 ns for **2** with an oxygen-saturated atmosphere. In Figure 3A, the kinetic traces at 500 nm of **1** (red) and **2** (blue) at different oxygen concentrations are depicted together with their monoexponential fits (compare with Table S2.2). The covariance matrix was used to estimate the uncertainties of the fit, yielding errors around 10%. For the first two components, we do not observe any oxygen dependence on the kinetics.

**Figure 3:**
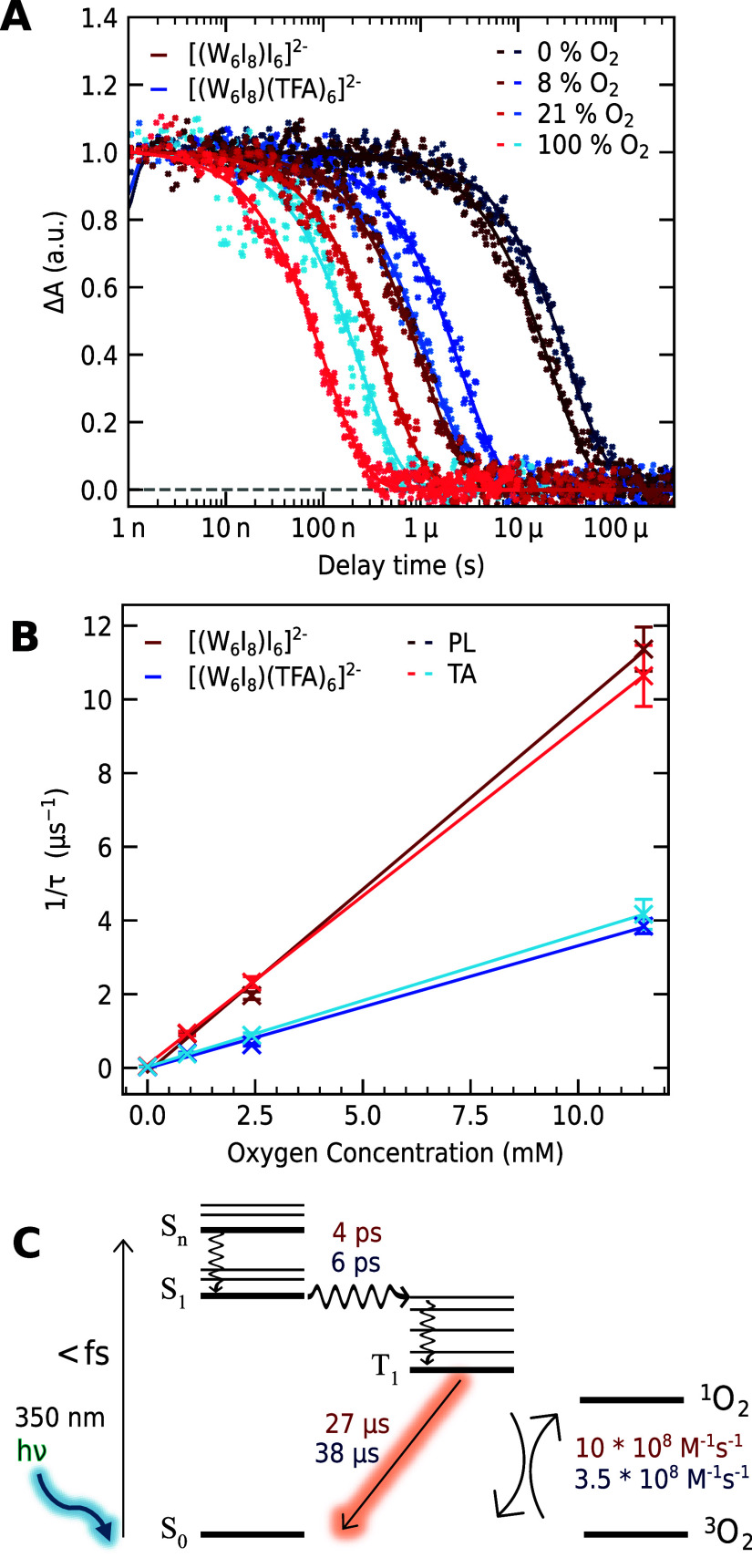
(A) Kinetic traces and monoexponential fits (straight) of ns-TA measurements of **1** (red) and **2** (blue) at 500 nm in acetonitrile and an excitation wavelength of 350 nm at room temperature. The different oxygen concentrations are indicated by the color gradient and range from 0% (argon, dark) to 100% (bright). (B) Oxygen concentration against the reciprocal monoexponential lifetime of **1** (red) and **2** (blue) measured by PL (dark) and TA (bright) spectroscopy. The linear fit is shown with a straight line. (C) Proposed Jabłoski diagram with ISC rates, emission lifetimes, and quenching rates of **1** (red) and **2** (blue) respectively.

We hypothesize that the third component corresponds to an emissive triplet state, which should exhibit similar kinetics in TAS and PL lifetime measurements. To test this, we conduct time-resolved photoluminescence as a third technique using the same oxygen concentrations as for the ns TA experiments (compare S3). As detailed in Figure 3B, the inverse lifetimes as a function of the oxygen concentration in both experiments are in good agreement, which supports our hypothesis. The linear relation between 1/τ and the oxygen concentration hints to a dynamic collisional quenching, whose bimolecular quenching constant *k*_q_ can be determined using eq 1:

$$\frac{1}{\tau }\propto k_{q}\times [O_{2}]$$ 1

where τ is the triplet lifetime at a given triplet oxygen concentration [O_2_] and *k*_q_ is the dynamic quenching constant. An oxygen concentration of 2.42 mM in acetonitrile exposed to synthetic air was used.^60^ The values are summarized in Table 1 and are in accordance with published work using emission spectroscopy to derive the quenching rates.^17,19^ The deviations of the lifetimes and subsequently the bimolecular quenching constants are within the error estimates of 10% for the TA experiments and 5% for the PL measurements, respectively. We assume that most of the deviation originates from uncertainties in setting and maintaining the oxygen concentration for and during the experiment, especially since TA experiments take significantly longer than corresponding PL experiments (h vs min).

The findings of the TA and PL verify the previous assumption that the third component observed in the TA experiments is the emissive triplet state and lead us to the conclusion that the second component is an excited singlet state. Following this reasoning, both clusters would undergo an ultrafast ISC into a long-lived triplet state within 6 ps. This is in accordance with our recent work on a related cationic tungsten iodide cluster, where we found an ISC occurring within 2.2 ps.^26^ In that study, we found an additional excited state decaying within 90 ps, which we could not observe in the current study.

The electronic transitions and lifetimes are summarized in a Jabłoski diagram in Figure 3C. The observation that the clusters relax to their final excited state (third component) within picoseconds with no significant deactivation occurring over 6 orders of magnitude is remarkable. This behavior accounts for their high phosphorescence quantum yields and pronounced sensitivity to oxygen. Note that these systems experience strong spin–orbit coupling, making singlet and triplet states lose their strict identity. It would therefore be more precise to describe these as mixed states with, e.g., a dominant singlet or triplet character.

Kirakci et al. investigated, with similar experimental conditions, molybdenum clusters and also found three components with comparable lifetimes and spectral shapes.^24^ They assigned the first component to an excited singlet state and the second component to a hot triplet state, relaxing to the third emissive component. This contrasts with our observations that the first component mainly originates from the solvent response and coherent artifacts close to the resolution limit. However, to this point, we cannot exclude the possibility that these features overshadow additional components close to our IRF.

Within experimental uncertainty, the temporal and spectral evolution of the ultrafast TA signals is comparable for both clusters studied and closely matches that reported for the cationic tungsten iodide cluster and molybdenum-based analogues.^24,26^ In all cases, the spectrum is dominated by a broad and unstructured ESA, revealing a multitude of contributing electronic states superimposed with ground-state bleaching, in line with steady-state absorbance data. The only significant difference is a modest variation in oscillator strength imposed by the axial ligands, highlighting that the ultrafast photophysical properties are mainly driven by the M_6_X_8_ core.

### T-Dependent Emission Characteristics in Solution

To further investigate the excited-state properties of the emissive triplets, we performed a series of temperature-dependent measurements from 4 to 340 K. At every temperature step, besides an emission spectrum, lifetimes were measured at least at 5 different emission wavelengths and globally fitted to increase the stability of the fit (see the Experimental Section for more details). Although measurements in solution involve a more elaborate sample preparation, more scattered light in frozen solvents, and a phase transition, they should reflect the fundamental photophysics better than polymer matrices or microcrystalline samples due to their well-defined and uniform properties. In acetonitrile, we found that below the melting point, the kinetics for **1** consistently changed from a mono- to a biexponential decay with lower overall lifetimes (see Figure S4.1). In preliminary experiments, we identified that the addition of (TBA)I reversed this and led to a longer and monoexponential decay in the solid state. This hints to a dynamic exchange reaction in solution and a disturbed and eventually frozen equilibrium while falling below the melting point, which is beyond the scope of this study (see S4 for a more detailed discussion).

In the following study in PMMA, we did not observe this behavior.

### T-Dependent Emission Properties in PMMA

The temperature-dependent emission spectra and lifetimes for both clusters in PMMA are shown in Figure 4, with color-coded temperature steps ranging from 4 K (dark) to 340 K (bright). At each step, an emission spectrum and multiple lifetimes were measured. In the emission spectra in Figure 4A, an asymmetrical broadening can be observed for both clusters upon increasing the temperature (compare FWHM values in Table 2 and Figure S5.1). However, a significant shift of the emission maximum can only be determined for **2.** Here, the maximum shifts from 4 and 662 to 675 nm toward 60 K and subsequently remains constant up to 340 K. In Figure S5.1, the emission maxima and FWHM are displayed against the temperature.

**Figure 4:**
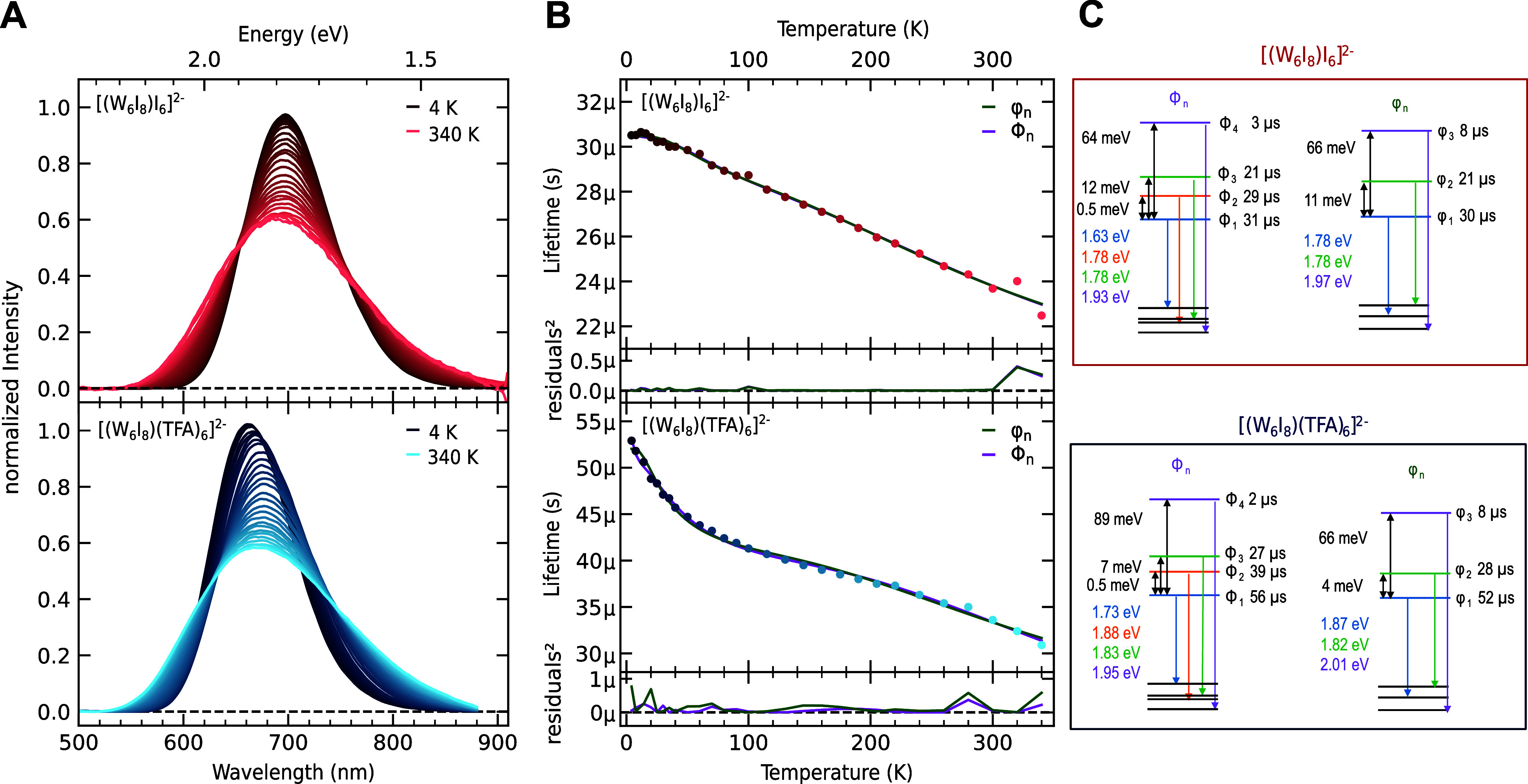
(A) Area-normalized intensity spectra of **1** (upper panel) and **2** (lower panel) at different temperatures ranging from 4 K (dark) to 340 K (bright). (B) Temperature dependence of the extracted lifetimes. At each temperature step, the time-resolved decay curve was fitted independently with a monoexponential model to yield lifetimes, which are plotted as a function of temperature. The colors of the dots correspond to the colors in panel (A). The straight line shows the best fit according to the Φ_n_ (purple) and φ_n_ (green) model, respectively. The squared residuals for both fits are shown below the plots. (C) Schematic representation of the extracted lifetimes and zero-field splitting according to both models and eq 2, as well as the emissive relaxation energies, according to eqs S6.1–4.

**Table 2:** T-Dependent Photophysical Properties of **1** and **2** in PMMA

compound		Δλ_max_, _4–300 K_	FWHM_4K_/meV	ΔFWHM_4–300 K_	τ_4K_/K	Δτ_4–300 K_	φ_n_ model ΔE_1n_/meV		Φ_n_ model ΔE_1n_/meV		
							*n* = 2	*n* = 3	*n* = 2	*n* = 3	*n* = 4
1	(TBA)_2_[(W_6_I_8_)I_6_]	<1%	243	44%	30	20%	11	66	0.5 (fixed)	12	64
2	(TBA)_2_[(W_6_I_8_)(TFA)_6_]	2%	254	43%	53	38%	4	66	0.5 (fixed)	7	89

The resulting lifetimes are shown in Figure 4B. For **1**, we find a monotonous, quasilinear decrease in the lifetime. In contrast, **2** features a sharp decrease in lifetime within the first 60 K, followed by an inflection point at around 150 K and subsequently a more gradual decrease (Table 2). It is noticeable that the dynamics found in the emission spectra are also reflected in the lifetime changes. While the spectral and kinetic evolution of **1** shows a more continuous course, the prominent emission shift upon increasing the temperature from 4 to 60 K for **2** correlates with a sharp decrease in the lifetime.

Especially for **1**, the emission and lifetime changes as a function of temperature appear to be small. From 4 K to room temperature, the relative lifetime change Δτ_4–300 K_ is 20% for **1** and 38% for **2**. Kitamura and co-workers found lifetime changes in a crystalline (TBA)_2_[(W_6_Cl_8_)­Cl_8_] sample from 42 μs at 3 K to 8 μs at 300 K, which results in a Δτ_4–300 K_ of 81%.^31^ Although high-resolution, temperature-dependent emission and lifetime measurements of tungsten clusters are scarce, related molybdenum clusters have been studied in more detail.^31–34^ Temperature-dependent emission measurements of [(Mo_6_I_8_)­(n-C_3_F_7_COO)_6_]^2–^ revealed a comparable temperature sensitivity with a Δτ_4–300 K_ of 31%.^33^ In a follow-up study,^32^ the influence of the inner and *apical* ligands of [(Mo_6_X_8_)­L_6_]^2–^ has been investigated further. In the series of [(Mo_6_X_8_)­I_6_]^2–^, the temperature sensitivity of the lifetime decreased gradually from Δτ_4–300 K_ = 78% for X = Cl, over 70% for X = Br, to 55% for X = l. In contrast, for (Mo_6_X_8_)­Br_6_ or a variation of the *apical* ligands, a trend could not be found. Although the authors identified a correlation between the properties of the ligands and the strength of the energy splitting of the triplet spin sublevels, no systematic change in the intrinsic emission lifetimes of these sublevels could be discerned. Given that the observed thermally equilibrated monoexponential lifetime is dependent on both the population and intrinsic emission lifetimes of the contributing spin sublevels, a prediction of the measured equilibrated lifetimes is not trivial.

### T-Dependent Emission Lifetime Model in PMMA

As detailed in the Introduction section, the symmetry-based spin-sublevel model was successfully applied for various molybdenum cluster complexes and explained systematic changes of the zero-magnetic-field splitting as a consequence of a change in electron density at the metal center.^32,34^ In the following, we fit and simulate our data with the proposed φ_n_ model using three triplet sublevels due to SOC and the Φ_n_ model using four nondegenerate sublevels due to a combination of SOC and symmetry lowering due to Jahn–Teller distortion. For the lifetime fit, we assume that every emitting level is in a Boltzmann equilibrium at any temperature. Hence, the overall lifetime as a function of temperature can be described as a sum of thermally activated individual rates as expressed in eq 2:

$$\tau (T)=\frac{\sum_{n}g_{n}e^{\frac{-\Delta E_{1n}}{k_{b}T}}}{\sum_{n}\frac{g_{n}}{\tau_{n}}e^{\frac{-\Delta E_{1n}}{k_{b}T}}}$$ 2

where *g*_*n*_ and τ_*n*_ are the degrees of degeneracy (see Table S6.1) and emission lifetimes of the sublevel φ_n_ or Φ_n_, respectively, and Δ*E*_1*n*_ is the energy difference between φ_1_ → φ_n_ or Φ_1_ → Φ_n_ (Δ*E*_11_ = 0). Furthermore, the Φ_n_ model predicts a constant energy difference Δ*E*_12_ of 4 cm^–1^ or 0.5 meV due to Jahn–Teller distortion.^32^

In Figure 4B, the results of the fitting of the φ_n_ (green) and Φ_n_ models (purple) are shown. We were not able to fit the data with only two components. Statistically, both models describe the temperature dependence of **1** equally well with a coefficient of determination (*r* ^2^) of 0.994 for the φ_n_ model and *r*_Φ_n__^2^ = 0.995. This is also found in the square of the residuals below the plot, showing no significant improvement of the Φ_n_ model over φ_n_. The additional degree of freedom of the Φ_n_ model is compensated by the small difference in lifetime of Φ_1_ and Φ_2_ of only 6%. For **2**, we find a minimal improvement of the fit noticeable in the residuals below 20 K with coefficients of determination of *r*_φ_n__^2^ = 0.995 and *r*_Φ_n__^2^ = 0.998. In the literature on molybdenum clusters, *r*_Φ_n__^2^ values between 0.982 and 0.999 are calculated.^32,33^ This indicates that our data and fit quality are comparable, although the lifetimes of tungsten clusters are normally about 1 order of magnitude faster than equivalent molybdenum clusters. Just based on the τ­(*T*) data, a statistical justification of four sublevels instead of three is questionable, especially for **1**. Based on the Bayesian information criterion (BIC), the φ_n_ model is slightly preferred for **1**, and the Φ_n_ model is preferred for **2**. However, potential systematic errors in temperature measurements, which are hard to model, may become particularly significant at lower temperatures. Based on a more elaborate analysis of the posterior probability distribution of the parameter space (see S5), the absolute values of the energy splitting and lifetimes should be considered as rough estimates due to correlations between sublevel splitting and the corresponding lifetime.

The extracted lifetimes and energy differences of the sublevels for both clusters and models are summarized in Figure 4C and Table 2. The variability of the fits can be found in Table S5.1. Note that the provided relaxation energies from the excited states to the relaxed ground states are based on the evaluation of the temperature-dependent emission spectra in Figure 5. The sublevel splitting therein is consistent with published work on [(Mo_6_I_8_)­l_6_]^2–^ (Δ*E*_13_ = 8.7 meV, Δ*E*_14_ = 91 meV) and [(W_6_Cl_8_)­Cl_6_]^2–^ (Δ*E*_13_ = 5 meV, Δ*E*_14_ = 77 meV).^31,32^ By introducing an electron-withdrawing ligand with trifluoroacetate, the Φ_n_ model predicts an increase in the zero-magnetic-field splitting, which we could observe for Δ*E*_14_, but not for Δ*E*_13_.

**Figure 5:**
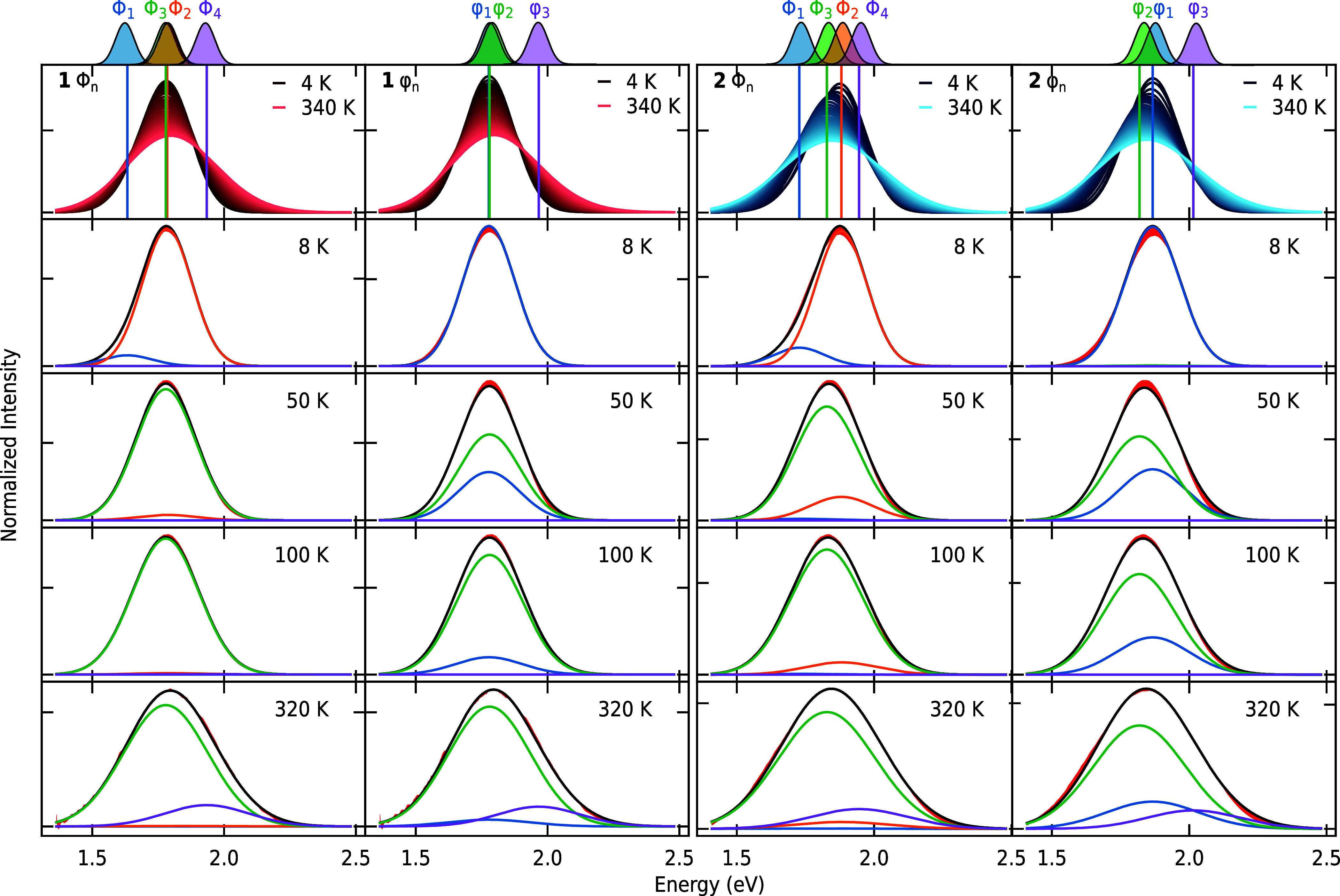
Simulated and observed temperature-dependent emission spectra of **1** (first two panels) and **2** (third and fourth panel) based on Φ_n_ (left) and the φ_n_ model (right). The energy positions of the states are shown in the first row. The spectral contribution of each sublevel to the overall fit (straight black line) is shown at selected temperatures. The residuals between the experimental data and the fit are highlighted in red.

### T-Dependent Emission Spectrum Model in PMMA

Based on the applied model, each sublevel contributes to the overall emission with an intrinsic emissive rate, emission maximum, and FWHM. At low temperatures, only the lowest sublevels should be populated, while with increasing temperature, the higher lying states become thermally accessible. Additional information is given in S6. We tested this model on the emission spectra of Figure 4A by applying the calculated zero-magnetic-field splitting of the sublevels from the lifetime measurements. The FWHM for every sublevel is kept constant at a given temperature, and a homogeneous Doppler broadening (∝ √*T*) is assumed.

Figure 5 shows the results of the intensity simulations according to equations S6.1–4. The first two columns show the fits of **1** using the Φ_n_ model with four sublevels and the φ_n_ model using three sublevels, respectively. The same is displayed for **2** in the third and fourth column. The emission maximum of each sublevel *E_n_* is depicted in the first row, and their corresponding spectra, as well as the envelope (straight black line), are displayed. When comparing the residuals between experimental and modeled data (highlighted in red), we find that both models can describe the emission data of **1** and **2** very well (*r* ^2^ < 0.996). At temperatures below 50 K, the additional splitting in the Φ_n_ model leads to a small improvement, which is most noticeable for **2**. Note that the lowest-lying sublevel Φ_1_ only leads to a small correction at temperatures below 8 K (first column and third column, blue component), which compensates the additional parameter in the Φ_n_- over the φ_n_ model. This observation is consistent with the lifetime fits in Figure 4, where we found no or only a slight improvement with the Φ_n_ model.

The fitting results are depicted in Figure 4C and Table S5.2. A detailed statistical analysis of the fitted models can be found in S5.

Based on these results, we find no clear experimental evidence that more than three energetically nondegenerate states are present for **1** and **2**, which is in line with the low-temperature investigations of crystalline (TBA)_2_[(W_6_Cl_8_)­Cl_6_].^31^ We conclude that the additional spitting of the lowest-lying state (e.g., T_2u_ {O_h_} → E_u_+B_2u_ {D_4h_}) predicted by the Φ_n_ model is not required to describe the temperature-dependent emission data of octahedral W­(II) clusters. To further interpret these findings, we performed TDDFT calculations of **1**, which will be discussed in the following section.

### TDDFT Computations

Although the φ_n_/Φ_n_ models include SOC and possible deformations, they are based only on group-theoretical symmetry considerations, resulting in a fine-structure splitting of the triplet states.

We performed TDDFT geometry optimizations, including scalar relativistic effects and SOC of the ground state (“SOC0”) and the three lowest-lying excited states (“SOC1–3”) based on a hybrid density functional that mixes PBE with Hartree–Fock exchange (see the Experimental Section for more details). In Figure 6, the calculated absorbance spectrum with the underlying transitions of **1** from the geometry-optimized SOC ground state SOC0 is shown, as well as the steady-state absorbance spectrum from Figure 1. We determine good agreement between our theoretical calculations, including relativistic SOC, and the experimental data.

**Figure 6:**
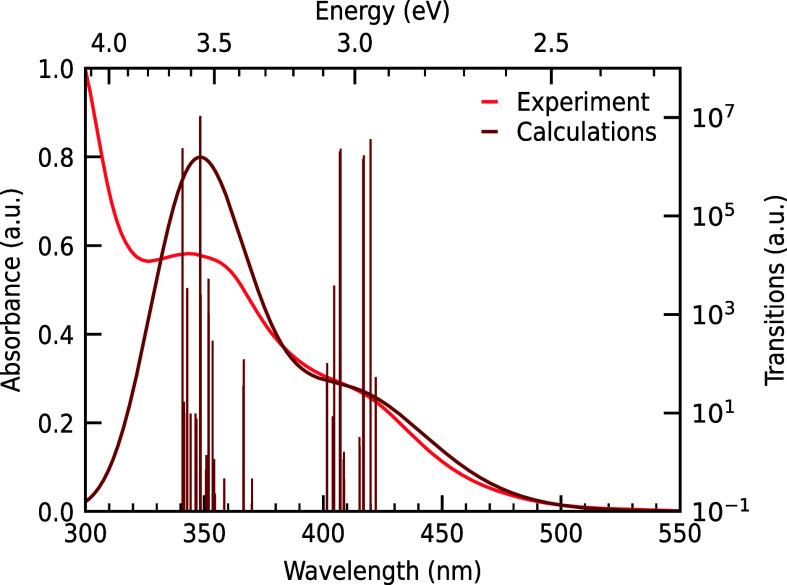
Calculated absorbance spectrum, requesting 50 states of **1** with a 0.5 eV line width from SOC0 geometry (dark). The underlying transitions (200 SOC transitions) and the experimental absorbance spectrum in acetonitrile (bright) are shown for comparison.

As a result of the geometry optimization of the lowest excited SOC states, we found significant distortions in the geometry relaxations, which are presented in Chart 3 and Table S7.9. Here, the SOC0 (W = red, I = yellow) and SOC1 (W = purple, I = green) geometries of **1** are depicted. We found that the symmetry of the O_h_ breaks by an outstretching of one W–I axis, accompanied by a movement of two adjacent inner iodide ions and a subtle twisting of the whole metal core. This is in line with TDDFT calculations on [Mo_6_Br_14_]^2–^ by Cordier and colleagues,^3^ who found a deformation of the metal core in the lowest triplet states, resulting in a similar symmetry reduction (C_2v_, C_s_, C_1_). The optimized SOC2 and SOC3 geometries can be found in Chart S7.1. Note that this reduction in symmetry to C_2v_ or even C_s_ for SOC2 would already mean a complete loss of degeneracy of the initially well-defined triplet states involved in the φ_n_/Φ_n_ models.

**Chart 3:**
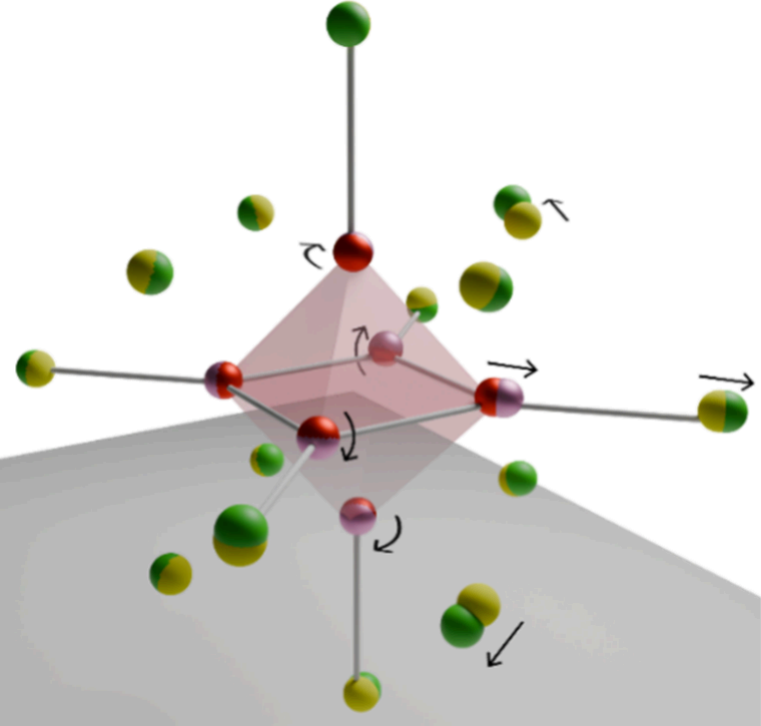
Distortions Found in the SOC1 Geometry (W = Purple, I = Green) Compared to SOC0 Geometry (W = Red, I = Yellow)

We report the sublevel energies of the SOC ground state and SOC1–3 geometries, as well as their corresponding mixture of the nonrelativistic basis states in Chart 4 and Table S7.1–4. We find that the three lowest excited states of each SOC geometry have mainly triplet character, while from the fourth level onward, significantly more states mix in. The theoretical calculations reveal that the energy barrier for the fourth substate (350 meV) is too high to allow a significant thermal population even at room temperature (*E*_rt_ ≅ 26 meV). These results support the luminescence data of both clusters, where only three distinct states were observed. The calculated relaxation energies from the respective excited-state geometry to the ground state and energy splitting within a single excited-state geometry are consistent with experimental data and fitted results, showing values of approximately 2 eV compared to 1.8 eV, and 4 and 16 meV compared to 12 and 66 meV.

**Chart 4:**
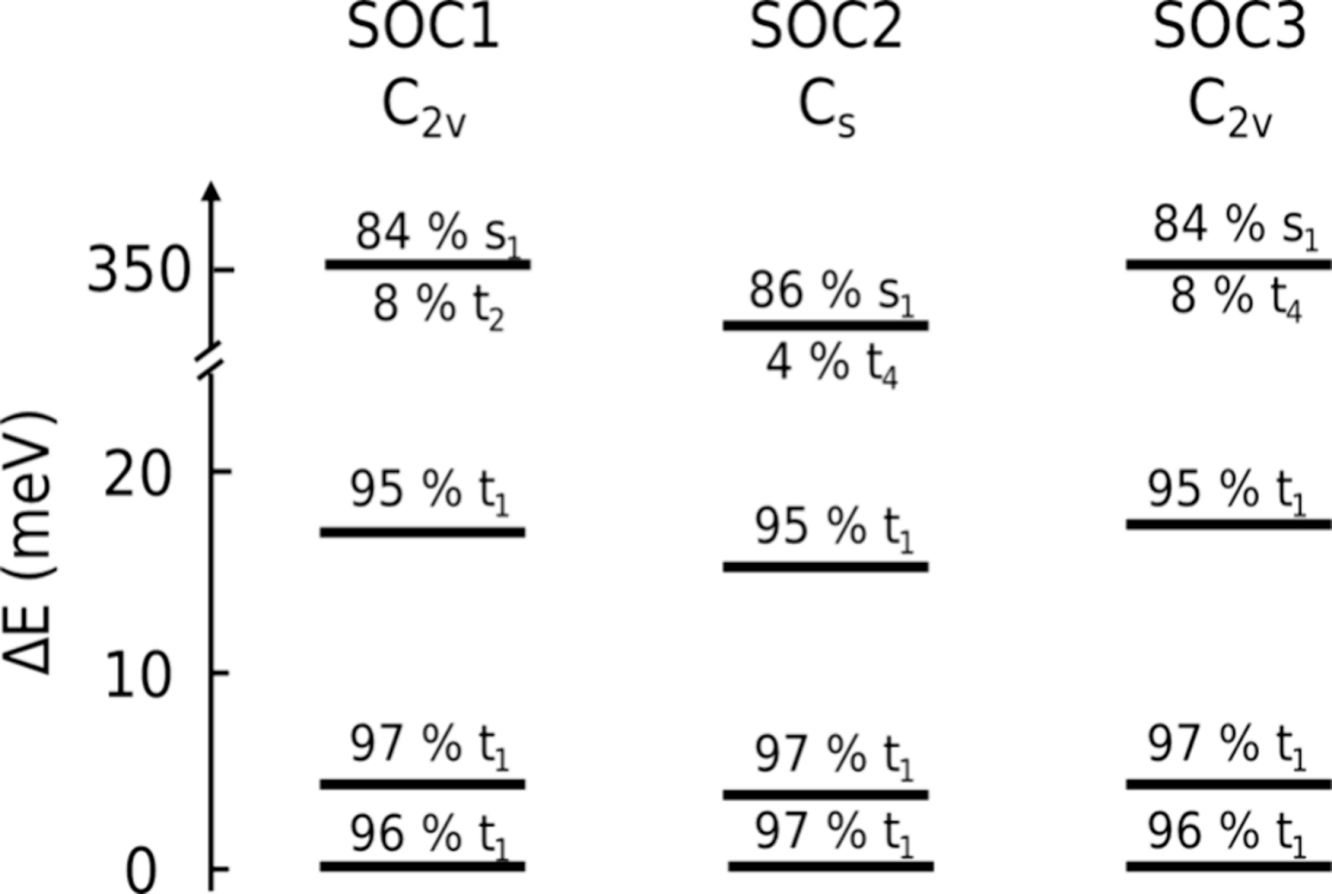
Sublevel Splitting of the Three Lowest-Lying SOC Geometries According to TDDFT Calculations

We note that all three SOC geometries are close in energy, composition, and sublevel splitting, which is in accordance with TDDFT calculations on [Mo_6_Br_14_]^2–^.^3^ Therefore, they can all contribute to the emission of the cluster, leading to a total of nine emissive states. Although these nine states are theoretically distinct and not required to be degenerate by symmetry, their energy difference can be too small to be probed individually even at 4 K, leading to a pseudodegeneracy.

Preliminary nonrelativistic calculations for **2** indicate a comparable symmetry reduction to that observed for **1** (see Chart S7.2 and Table S7.12).

## Conclusions

In this comprehensive study, we analyzed two tungsten iodide prototype clusters (TBA)_2_[(W_6_I_8_)­I_6_] (**1**) and (TBA)_2_[(W_6_I_8_)­(TFA)_6_] (**2**) using various time- and temperature-dependent experimental techniques. Ultrafast TAS reveals that both clusters undergo ISC within 6 ps, rapidly populating their relaxed triplet states. Subsequently, excited-state deactivation occurs via emission or dynamical bimolecular quenching through ^3^O_2_, which was traced by transient absorption and emission spectroscopy.

The temperature-dependent emission characteristics of both clusters in PMMA from 4 to 340 K can be satisfactorily modeled by three emissive sublevels, as predicted by the group-theoretical φ_n_ model. Unlike observations in molybdenum-based clusters, no evidence for an additional splitting of the lowest triplet sublevels was detected. In fact, TDDFT calculations suggest significant excited-state geometry distortion, questioning whether purely group-theoretical descriptions adequately capture the emission behavior in tungsten iodide clusters. According to the model presented here, emission from the initial 9-fold degenerate triplet manifold arises from three distinct excited-state geometries, each providing three accessible triplet states. These excited-state geometries are energetically close and thermally accessible, probably leading to pseudodegeneracy and the experimentally resolvable states. Consequently, subtle variations in ligand environment or cluster packing may influence the excited-state landscape, potentially lifting pseudodegeneracies and uncovering additional spin–orbit-coupled sublevels. This picture extends the Mo-based framework of Costuas et al., where at least two relaxed geometries contribute to the NIR luminescence, to tungsten iodide clusters in the methodological context of this study.^3^ However, direct quantitative comparison across models is limited due to different experimental and computational methods and the strong influence of the different metal centers and ligands. Alternative explanations based on diffusion-controlled kinetics, solvation, or vibrational cooling can be ruled out due to the rigid PMMA matrix used and the strictly monoexponential decay trace at each temperature.

Resolving these subtleties in the emissive triplet-state manifold is critical, not only for a deeper photophysical understanding but also for advancing the practical utility of tungsten iodide clusters in biomedical imaging and oxygen-sensitive therapeutic applications. Future investigations should thus correlate subtle structural variations directly with emission properties and TDDFT calculations to enhance the predictive design of cluster-based functional materials.

## Supplementary Material



## Notes

Transient absorption data of **2** and the pure solvent, oxygen-dependent emission lifetime spectra of **1** and **2**, t-dependent emission data of **1** in solution, t-dependent emission data of **1** and **2** in PMMA, additional information on the chosen models and fit statistics, and optimized SOC geometries and eigenvectors from the TDDFT calculations (PDF)

## Abbreviations

EADS: evolution-associated difference spectra
ESA: excited-state absorption
IRF: instrument response function
ISC: intersystem crossing
PMMA: poly­(methyl methacrylate)
PL: photoluminescence
SOC: spin–orbit coupling
TAS: transient absorption spectroscopy
TDDFT: time-dependent density functional theory
TBA: tetra-*n*-butylammonium
(TBA)­I: tetra-*n*-butylammonium iodide
TFA: trifluoroacetate
TRES: time-resolved emission spectra
WLC: white-light continuum
