# Supplementary material for: Time- and Temperature-Resolved Triplet Dynamics in Tungsten Iodide Clusters
Source: Inorg Chem. 2025 Oct 6;64(41):20584–95. doi: 10.1021/acs.inorgchem.5c02344 (PMC12541705; doi:10.1021/acs.inorgchem.5c02344)
Supplement: Supplementary file 1 [file ic5c02344_si_001.pdf]

Supporting Information for

Time- and Temperature-Resolved Triplet Dynamics in Tungsten Iodide Clusters

Philipp Frech,[1] Wolfgang Leis,[2] Florian Pachel,[3] Michael Seitz,[2] H.-Jürgen Meyer[3] and Marcus Scheele[1,*]

[1]Institute of Physical and Theoretical Chemistry, University of Tübingen, Auf der Morgenstelle 18, 72076 Tübingen, Germany

[2]Institute of Inorganic Chemistry, University of Tübingen, Auf der Morgenstelle 18, 72076 Tübingen, Germany

[3]Section for Solid State and Theoretical Inorganic Chemistry, Institute of Inorganic Chemistry, University of Tübingen, Auf der Morgenstelle 18, 72076 Tübingen, Germany

Mail: marcus.scheele@uni-tuebingen.de

Content

S1 Additional Experimental Information 1

S2 Transient Absorption Data 2

Analysis of **2** 2

Analysis of the IRF and Solvent Response 2

Fitting Model and Variability 3

Analysis of the long-lived Component with ns TA 4

S3 Emission Data at 295 K and Oxygen Quenching 5

S4 Analysis of the T-Dependent Emission Data of **1** in Solution 7

S5 T-Dependent Emission Data of **1** and **2** in PMMA 9

Variability of the Lifetime Fits 9

Fitting Results of the Spectral Emission Fits 13

Variability of the Spectral Emission Fits 14

S6 Additional Information on the φ_n/Φ_n model 19

Model Assumptions and Comparison: 19

Derivation of the spectral fit 19

S7 TDDFT Analysis 21

Optimized Geometries for the SOC2 and SOC3 states of **1** 21

Eigenvectors of the SOC0-3 matrices of **1** 21

Optimized Geometries for the S_0 and T_1 states of **2** 26

References 29

S1 Additional Experimental Information

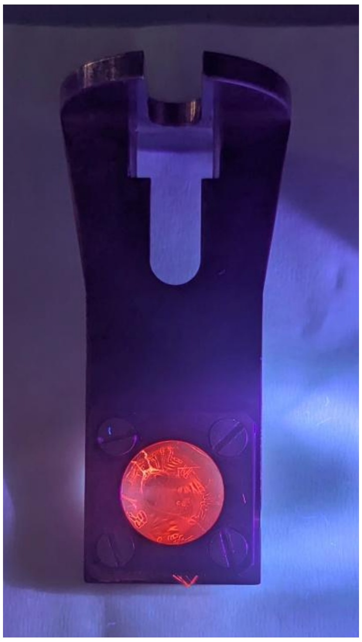

Figure S1.1. PMMA film of **1** under UV light.

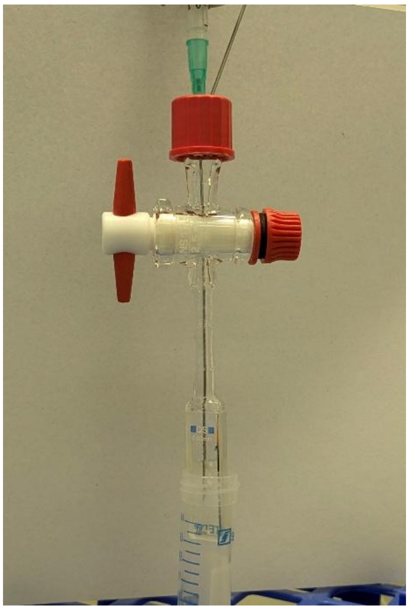

Figure S1.2. Microcuvette with homebuilt opening to control the atmosphere within the cuvette.

S2 Transient Absorption Data

Analysis of **2**

The analysis of the fs TA experiments of **2** in MeCN (λ_{exc} = 350 nm) are shown in Figure S2.1. The procedure is the same as described in the main manuscript. In Figure 3A the background corrected 2D hyperspectrum is depicted. A sequential fit at 500 nm (Figure S2.1B) required 3 components with lifetimes of 270 fs, 4 ps and a lifetime longer than our measurement window of 2 ns (r^2 = 0.99). Global fitting over the whole spectral range gave comparable lifetimes of 370 fs (red) and 5.6 ps (blue) and a coefficient of determination of 0.98. The corresponding EADS are shown in Figure S2.1C.

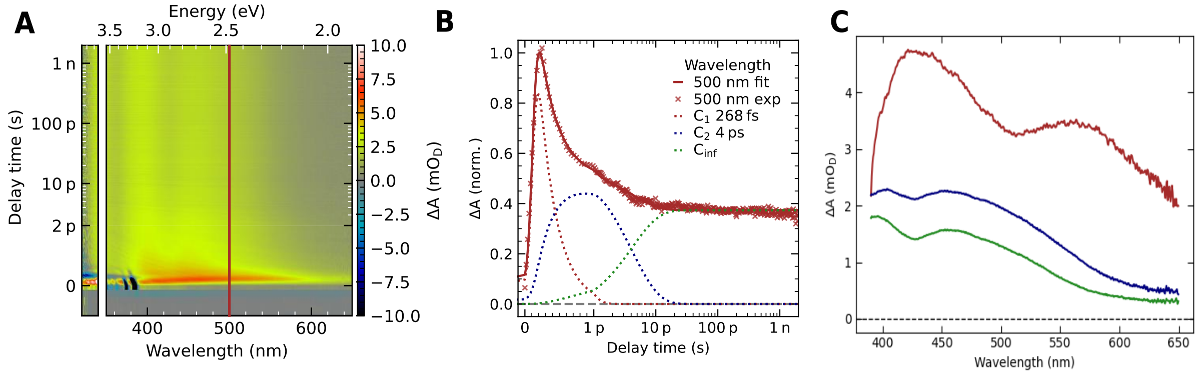

Figure S2.1. (A) fs TA-hyperspectrum of 2 in acetonitrile after excitation at 350 nm. The background is corrected to account for the overlying emission starting around 550 nm. The delay time between pump and probe beam within the first two ps are displayed with a linear scale followed by a logarithmic scale until the end of the measurement window of 2 ns. (B) Kinetic trace of (A) at 500 nm with the corresponding local exponential fit (straight) and the underlying three sequential exponential functions (dotted). (C) Evolution associated spectra of the global analysis of (A). Red corresponds to the first component, blue to the second and green represents the long-lived third component.

Analysis of the IRF and Solvent Response

Figure S2.2 presents the fs TA data of pure acetonitrile under the same experimental conditions as for the ultrafast experiments of **1** and **2**. In Figure S2.2A, the 2D spectrum is depicted while the kinetic trace at 500 nm as well as the fit is shown in Figure S2.2B. Here, we find a main component decaying with 90 fs and a second weak decay of 900 fs (r^2 = 0.96). The same results can be reproduced by global fitting giving lifetimes of 140 fs and 900 fs (r^2 = 0.91). The corresponding EADS with the first component in red and the second component in blue is shown in Figure S2.2C. Since MeCN does not absorb the excitation wavelength, we contribute these signals to the IRF, solvent response and coherent artifacts.

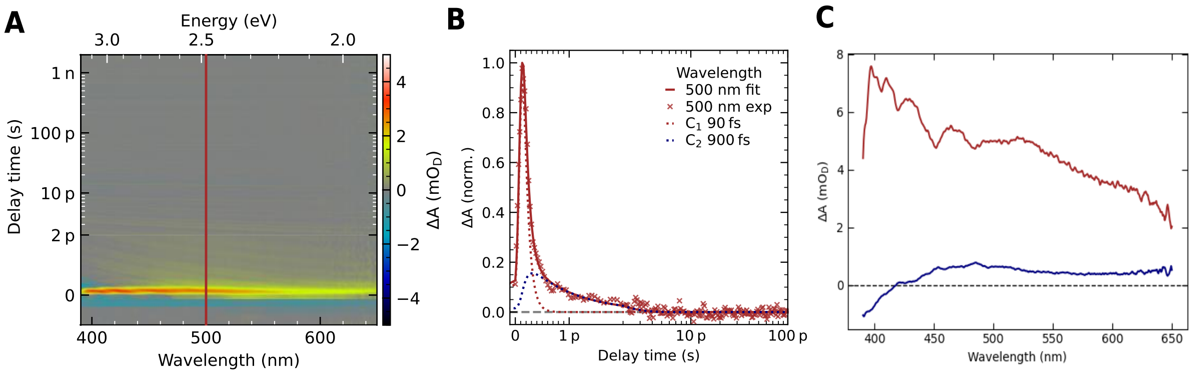

Figure S2.2. (A) fs TA-hyperspectrum of acetonitrile after excitation at 350 nm. The delay time between pump and probe beam within the first two ps are displayed with a linear scale followed by a logarithmic scale until the end of the measurement window of 2 ns. (B) Kinetic trace of (A) at 500 nm with the corresponding local exponential fit (straight) and the underlying three sequential exponential functions (dotted). (C) Evolution associated spectra of the global analysis of (A). Red corresponds to the first (140 fs) and blue to the second component (900 fs).

Fitting Model and Variability

Local and global fitting analysis was performed using a sequential model with three components A -> B -> C convoluted with the instrument response function (IRF). The IRF was modeled with a Gaussian function and C was set to a non-decaying constant term. Table S2.1 lists the statistical 1 σ uncertainties obtained from the Jacobian together with the Pearson correlation coefficients that most affect those uncertainties. Global fitting tightens the confidence intervals relative to the local fits, but strong anti-correlation between the IRF width and the fastest time constant τ_1 (up to –0.75) remains unavoidable because the IRF (~200 fs FWHM) is of the same order as τ_1. This coupling implies that the practical uncertainty of τ_1 is somewhat larger than its formal 1 σ value, whereas τ_2 is less affected. Accordingly, we estimate the uncertainty of the relevant ISC parameter τ_2 extracted from global analysis at a conservative 10 to 15%.

Table S2.1. Parameter uncertainties *and parameter-parameter correlations* of the *local (500 nm) and global fits of the TA data*.

Compound	fit	τ_1	τ_2	IRF- τ_1 correlation	τ_1 - τ_2 correlation
1 $(TBA)_2[(W_6I_8)I_6]$	local	55 %	15 %	-70 %	40 %
1 $(TBA)_2[(W_6I_8)I_6]$	global	12 %	6 %	-70 %	24 %
2 $(TBA)_2[(W_6I_8)(TFA)_6]$	local	23 %	30 %	- 60 %	55 %
2 $(TBA)_2[(W_6I_8)(TFA)_6]$	global	5 %	7 %	- 50 %	50 %
Acetonitrile	local	8 %	11 %	- 75 %	55 %
Acetonitrile	global	4 %	7 %	- 60 %	30 %

Analysis of the long-lived Component with ns TA

In Table S2.2, the monoexponential fits of the kinetic traces at 500 nm of Figure 3A of the main manuscript are shown.

Table S2.2. Monoexponential fits of 1 and 2 at different oxygen concentrations corresponding to Figure 3A from the main manuscript. Errors are estimated to be within 10 %.

Compound	τ(0 % O_2) / µs	τ(8 % O_2) / µs	τ(21 % O_2) / µs	τ(100 % O_2) / µs
1 $(TBA)_2[(W_6I_8)I_6]$	22	1.1	0.43	0.09
2 $(TBA)_2[(W_6I_8)(TFA)_6]$	33	2.7	1.13	0.24

S3 Emission Data at 295 K and Oxygen Quenching

In Figure S3.1 the emission lifetime traces of **1** and **2** in MeCN at different oxygen concentrations are depicted (λ_{exc} = 375 nm, λ_{em} = 680 nm). They were fitted monoexponentially giving lifetimes shown in Table S3.1. The corresponding emission spectra are shown in Figure S3.2.

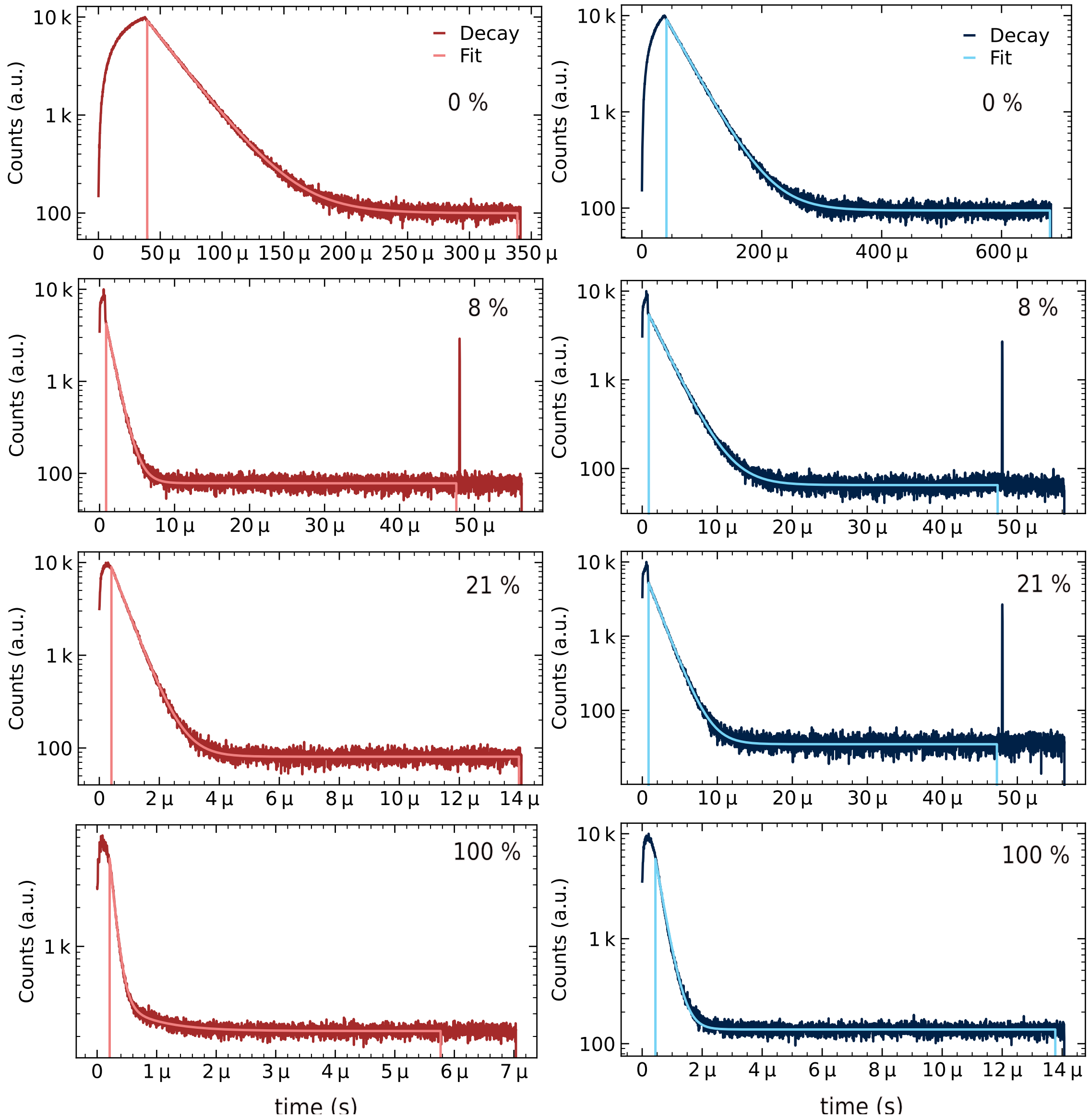

Figure S3.1. All emission lifetime traces + fits of **1** (right column red) and **2** (left column blue) at different oxygen concentrations.

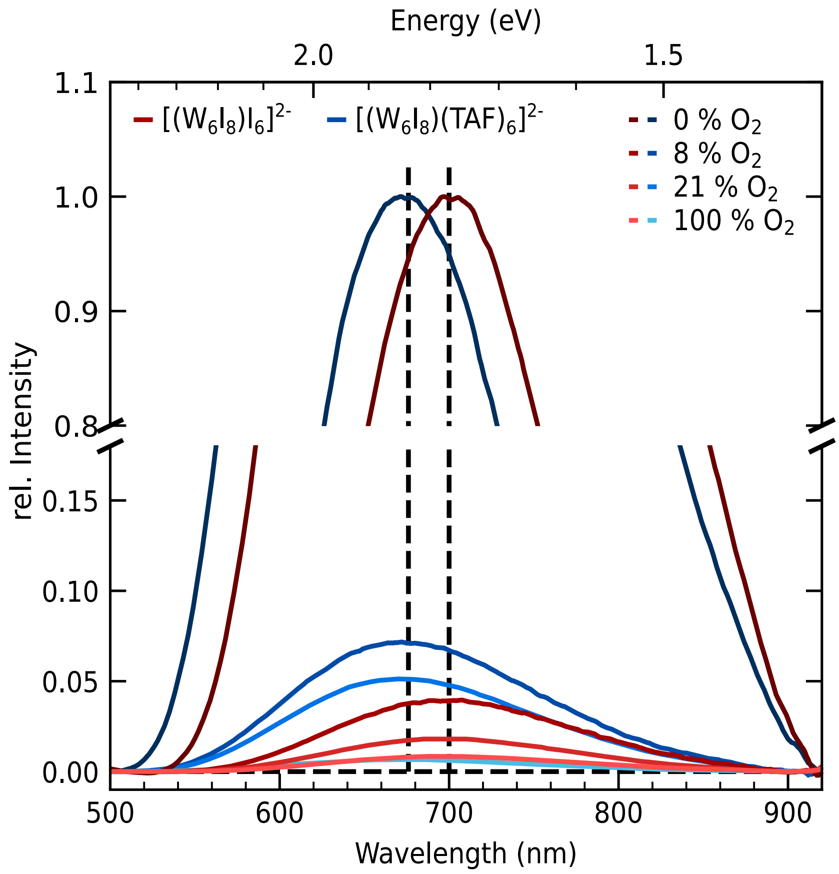

Figure S3.2. Emission Spectra of **1** and **2** at different oxygen concentrations.

Table S3.1. Monoexponential lifetime fits of 1 and 2 at different oxygen concentrations. Errors are estimated to be within 5 %.

Compound	τ(0 % O_2) / μs	τ(8 % O_2) / μs	τ(21 % O_2) / μs	τ(100 % O_2) / μs
1 $(TBA)_2[(W_6I_8)I_6]$	26.8	1.08	0.51	0.09
2 $(TBA)_2[(W_6I_8)(TFA)_6]$	38.67	2.49	1.66	0.26

S4 Analysis of the T-Dependent Emission Data of **1** in Solution

In Figure S4.1, the lifetimes and emission spectra of **1** in MeCN as a function of temperature are displayed as well as the phase transition at 228 K with a dashed line. In Figure S4.1A, the lifetimes were fitted with two components showing a clear biexponential decay in the solid state which transitions to the expected monoexponential decay above the melting point. The emission spectra in Figure S4.1B show an emission maximum of 700 nm in the liquid state and a maximum centered at 685 nm when frozen. Beside that shift of 40 meV at the phase transition, the spectral characteristics resemble the dynamics found in a PMMA matrix. Although a blueshift of the emission maximum can be expected due to a decreased stabilization of the triplet in the solid state, the drastic changes in the emission lifetime are unlikely to only originate from the different state of matter. A possible explanation could be a dynamic equilibrium which is disturbed and eventually frozen while falling below the melting point. Especially the *apical* ligands are only weakly bound to the $[W_6I_8]^{4+}$ core and can be exchanged easily with silver carboxylates under the formation of AgI.[1,2] Recently, we synthesized the cationic $[(W_6I_8)(CH_3CN)_6]^{4+}$ and $[(W_6I_8)I(CH_3CN)_5]^{3+}$, proving that acetonitrile can also stabilize the cluster with a suitable counter ion.[3] Although $(TBA)_2[(W_6I_8)I_6]$ would be the thermodynamic stable configuration, $(TBA)[(W_6I_8)I_5(CH_3CN)]$ + (TBA)I or even $[(W_6I_8)I_4(CH_3CN)_2]$ + 2 (TBA)I could be kinetically trapped at the phase transition leading to a drastic quenching of the lifetimes.

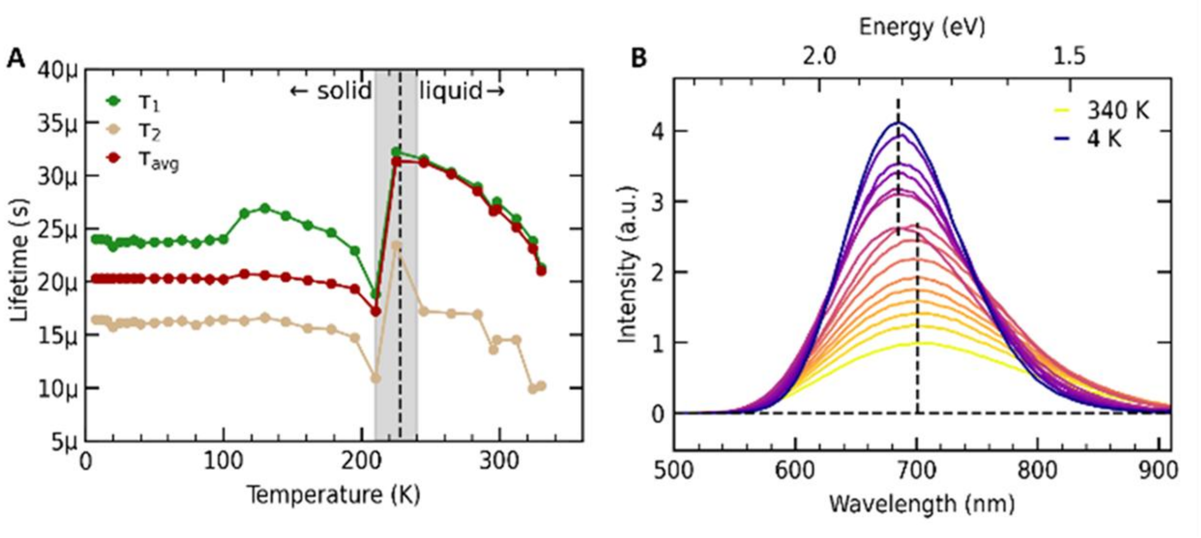

Figure S4.1. (A) Biexponential and average lifetimes of **1** in MeCN extracted at different temperatures. The gray area represents the phase transition of MeCN at 228K. (B) Corresponding emission spectra at each temperature step. The dashed line marks the emission maximum.

This interpretation would have multiple consequences. First, the resulting biexponential lifetimes in the frozen state would change when changing the temperature gradient used to freeze the sample. Second, the addition of (TBA)I should reduce the biexponential behavior according to Le Chatelier's principle and third, the different

binding situation of TFA in **2** should also change the dynamic exchange reactions. Figure S4.2A shows the biexponential lifetime of **1** at room temperature and 77 K as a function of addition of (TBA)I. In the upper panel at room temperature, we found the expected monoexponential lifetime of 26 µs which is stable against small amounts of additional (TBA)I. If frozen with liquid nitrogen, we again find a biexponential behavior resulting in an overall lower average lifetime of 24 µs, although the lifetimes and amplitudes are slightly different compared to the experiments performed in a cryostat. Upon addition of large amounts of (TBA)I (e.g. 1000 : 1 or higher) at room temperature, we detect an increased quenching introduced by (TBA)I leading to a lifetime drop by 12 % to 23 µs. Besides that, we find no additional influence of (TBA)I at room temperature, which can also be seen in the emission spectra in Figure S4.2B. This changes drastically at 77 K. Here we find that the addition of (TBA)I (1000 equivalents) reduces the biexponential behavior and leads to an increase in lifetime. This is consistent with our hypothesis, that exchanged or missing *apical* iodide is **not** only the cause of the different dynamics observed below the melting point but also can be addressed with competing ligands. This is supported by the small changes in the emission spectra in Figure S4.2B at 77 K which point to a different composition of frozen cluster species because of different (TBA)I amounts.

In case of **2**, we find no significant biexponential character at 77 K in solution with a monoexponential decay of 40 µs, which only differs by 5% compared to the experiments in PMMA. This leads us to the conclusion that TFA^- is less susceptible to exchange reactions compared to I^-, probably due to its bidentate nature.

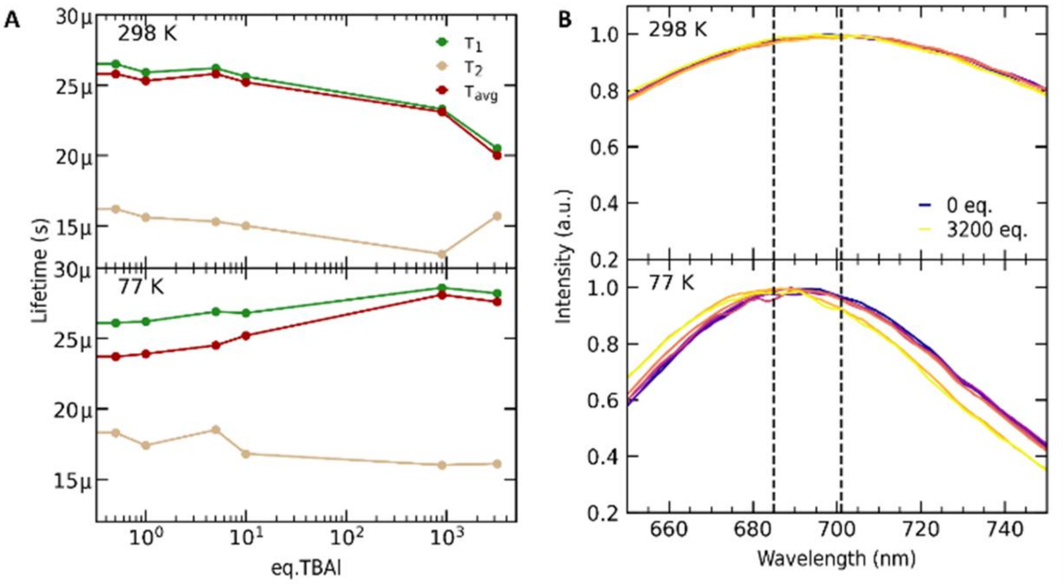

Figure S4.2. (A) Lifetimes of **1** in solution at room temperature (upper panel) and 77 K (lower panel) as a function of equivalents of (TBA)I. (B) Corresponding emission spectra

S5 T-Dependent Emission Data of **1** and **2** in PMMA

To extract the emission maximum and FWHM at every temperature step for **1** and **2**, a single Gaussian function was fitted at every temperature. Although this did not fit the emission well over the whole spectral range, especially at higher temperatures, it gives a systematic measure of the emission maximum and FWHM values. The results are shown in Figure S5.1

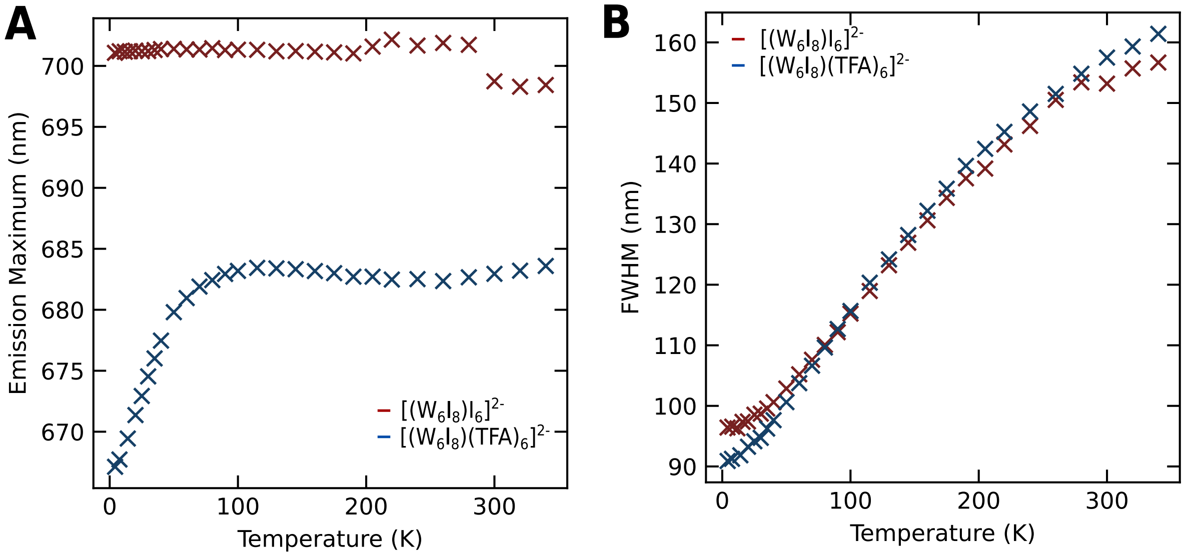

Figure S5.1. Emission maximum (A) and FWHM (B) of **1** and **2** at every temperature step extracted from Figure 4A from the main manuscript. The values were extracted by fitting a single Gaussian to each emission peak.

Variability of the Lifetime Fits

The relative parameter uncertainties of the fitted models are summarized in Table S5.1. They were estimated by the covariance matrices provided by the python module lmfit.

Table S5.1. Parameter uncertainties of the T-dependent lifetime fit estimated from the covariance matrices for **1** and **2**.

Model	Cluster	E_{12}	E_{13}	E_{14}	τ_1	τ_2	τ_3	τ_4
φ_n	**1**	22 %	23 %	-	1 %	9 %	25 %	-
Φ_n	**1**	fixed	24 %	22 %	1 %	4 %	9 %	20 %
φ_n	**2**	8 %	14 %	-	1 %	2 %	24 %	-
Φ_n	**2**	fixed	9 %	12 %	1 %	4 %	2 %	31 %

We used a python implementation of the Markov chain Monte Carlo (MCMC) called emcee[4] to study the posterior distributions of the fitting parameters and to extract correlations between those. Figure S5.2-5 shows the results of the MCMC study using a corner plot representation.[5] Here, the histograms on the diagonal axis show the

marginalized distribution of each parameter (uncertainty) and the standard deviation of the noise σ separately, while the other plots depict the joint distribution of a pair of parameters (correlation). We find negative correlations between the splitting of each sublevel and the corresponding lifetime for both models. This is especially true for the highest sublevel which is also reflected in the high uncertainty values in Table S5.1. This can be rationalized by the lack of datapoints above 340 K. In the ϕ_n model, we determine additionally high negative correlation between τ_1 and τ_2 mainly because of the proximity in energy and lifetime. We could not find a correlation between the noise level and parameter estimates. Based on this analysis, the values for the energy splitting and sublevel lifetimes should be understood as rough estimates especially for **1**.

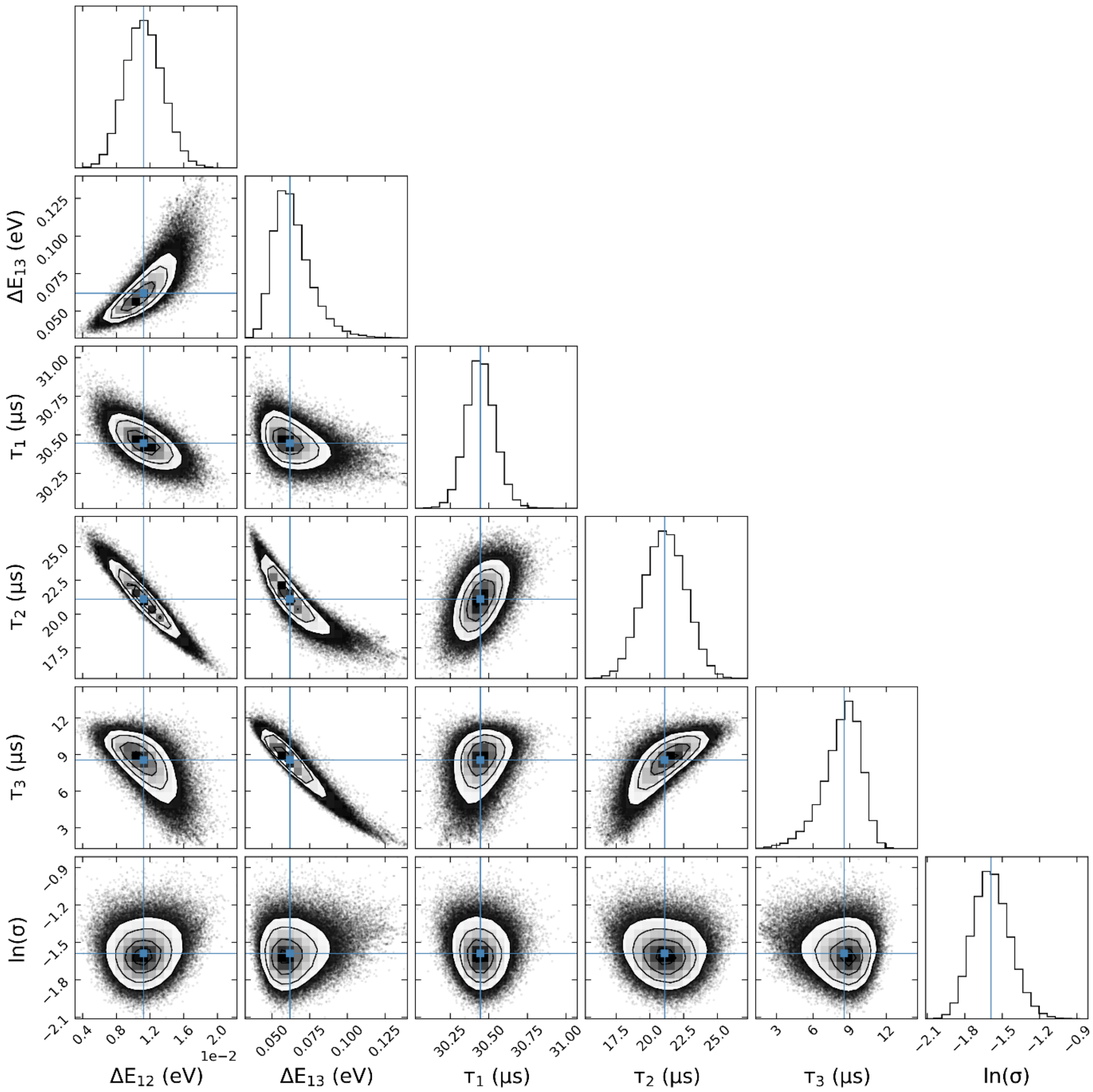

Figure S5.2 Corner Plots of the MCMC posterior parameter distribution of the lifetime fits of **1** using the φ_n model. On the diagonal axis the marginalized distribution (uncertainty) of each parameter including the noise σ is shown in a histogram while the other plots show the correlation of two parameter sets.

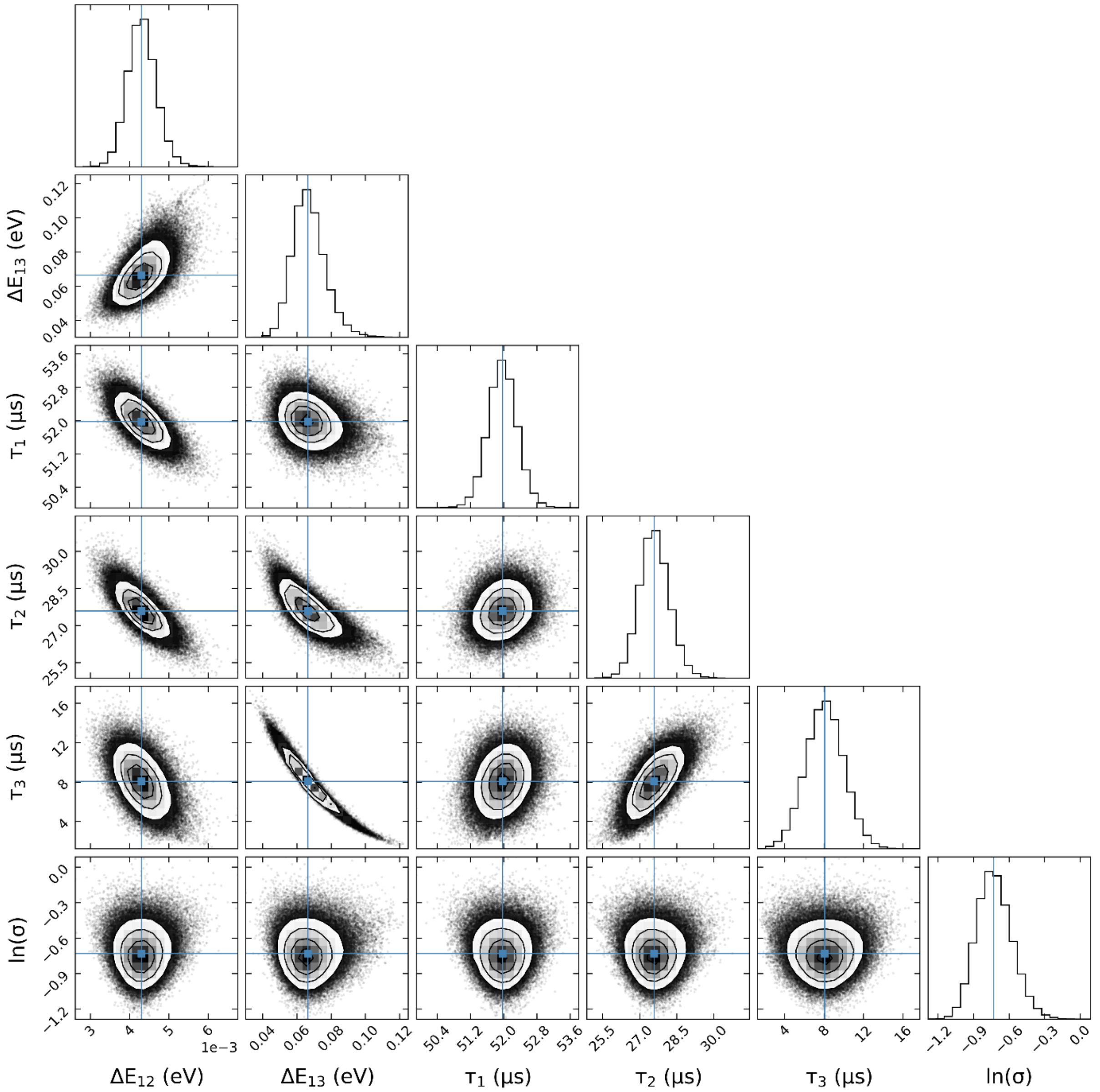

Figure S5.3 Corner Plots of the MCMC posterior parameter distribution of the lifetime fits of **2** using the φ_n model. On the diagonal axis the marginalized distribution (uncertainty) of each parameter including the noise σ is shown in a histogram while the other plots show the correlation of two parameter sets.

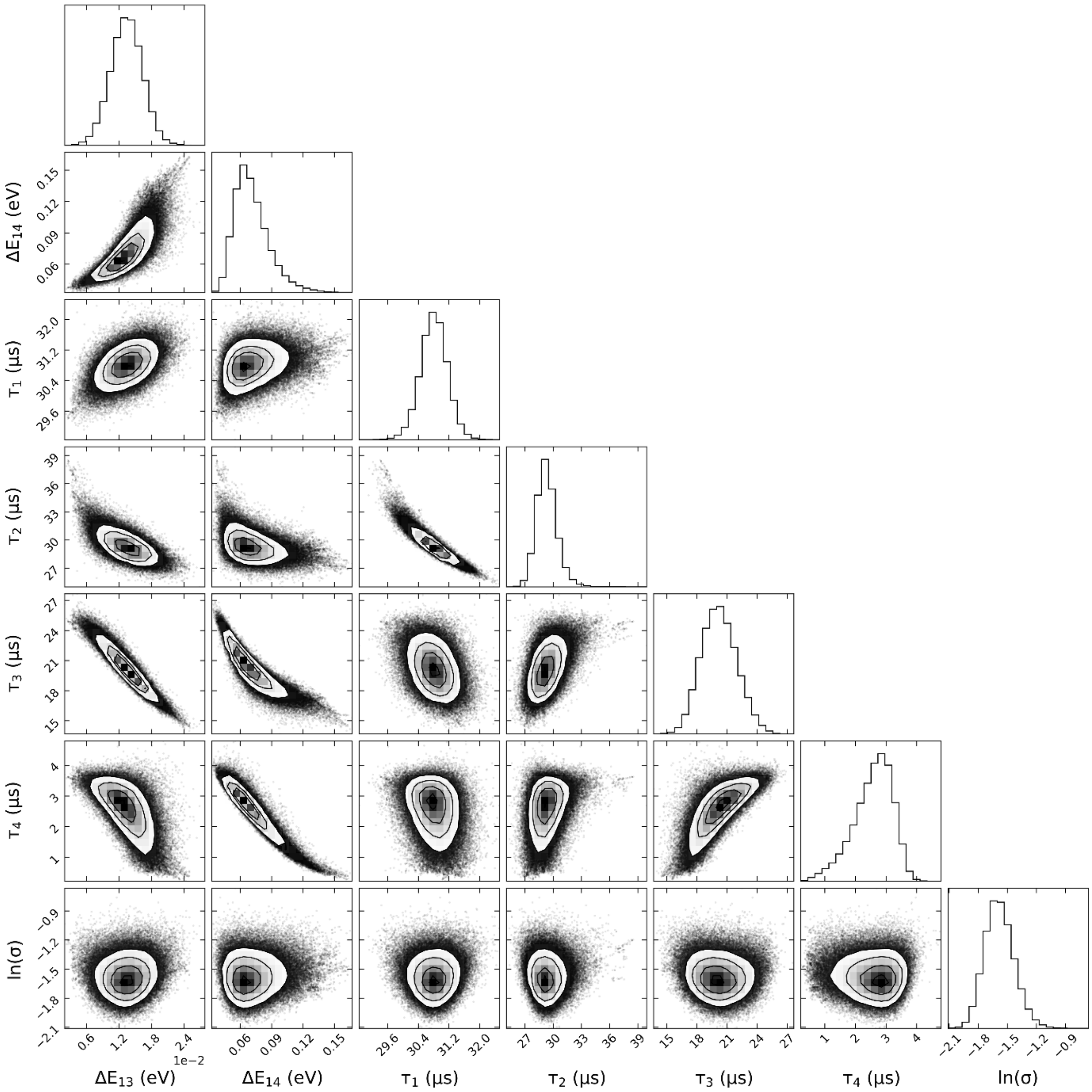

Figure S5.4 Corner Plots of the MCMC posterior parameter distribution of the lifetime fits of **1** using the ϕ_n model. On the diagonal axis the marginalized distribution (uncertainty) of each parameter including the noise σ is shown in a histogram while the other plots show the correlation of two parameter sets.

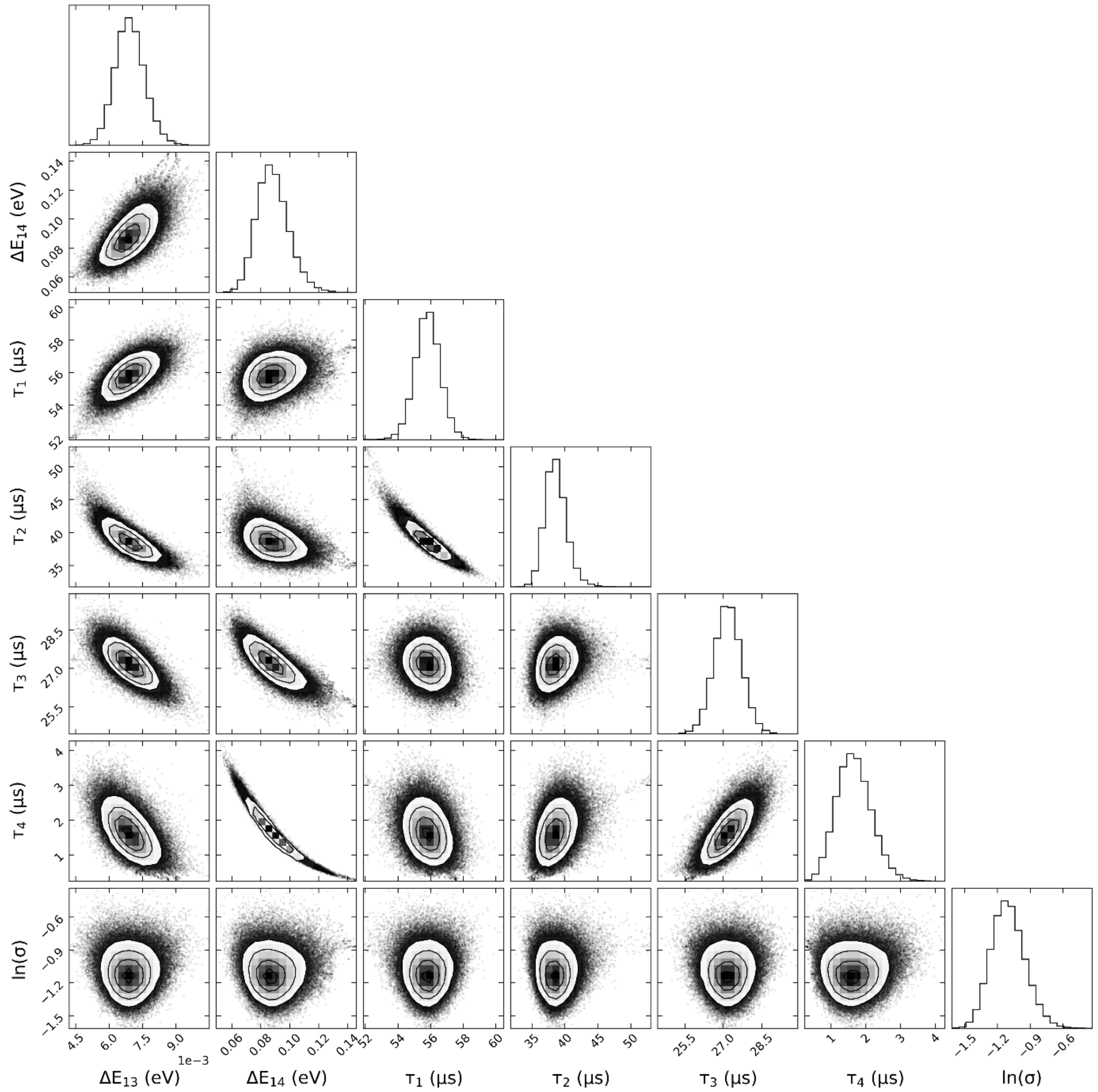

Figure S5.5 Corner Plots of the MCMC posterior parameter distribution of the lifetime fits of **2** using the ϕ_n model. On the diagonal axis the marginalized distribution (uncertainty) of each parameter including the noise σ is shown in a histogram while the other plots show the correlation of two parameter sets.

Fitting Results of the Spectral Emission Fits

Table S5.2. Fitting results of the T-dependent spectral measurements for **1** and **2**. Parameter names refer to equations S6.1-S6.4.

Model	Cluster	E_1 / eV	E_2 / eV	E_3 / eV	E_4 / eV	k_2 /Hz	k_3 /Hz	k_4 /Hz	$FWHM_0$[1] / meV	$k_{broadening}$[1] / meV
**1** φ_n	**1**	1.78	1.78	1.96	-	26	32	-	177	8
**1** Φ_n	**1**	1.63	1.78	1.78	1.93	26	10 000	11 000	165	9
**2** φ_n	**2**	1.87	1.82	2.01	-	4	7	-	177	9
**2** Φ_n	**2**	1.73	1.88	1.83	1.95	15	332	1 000	155	11

[1] $FWHM_0$ refers to the theoretical FWHM at 0K and $k_{broadening}$ to the proportionality constant of the Doppler broadening (see equation S6.4)

Table S5.3. Fitting results of the T-dependent spectral measurements for **1** and **2**. E_n refers to the emission maximum, and the $FWHM_{xK}$ represents the temperature-dependent broadening according to equation S6.4.

Model	Cluster	E_1 / nm	E_2 / nm	E_3 / nm	E_4 / nm	$FWHM_{4K}$ / meV	$FWHM_{77K}$ / meV	$FWHM_{200K}$ / meV	$FWHM_{300K}$ / meV
**1** φ_n	**1**	698	697	630	-	193	247	290	315
**1** Φ_n	**1**	759	695	697	641	183	244	292	321
**2** φ_n	**2**	664	682	615	-	195	256	304	333
**2** Φ_n	**2**	718	659	678	637	177	252	311	346

Variability of the Spectral Emission Fits

The fits of the models on the spectral emission shape are analyzed as above. The calculated uncertainties of every E_n value are smaller than 1 % while the difference between the radiative rates Δk_{1n} can have larger uncertainties. The relative parameter uncertainties are summarized in Table S5.4.

Table S5.4. Parameter uncertainties of the T-dependent spectral fit estimated from the covariance matrices for **1** and **2**.

Model	Cluster	$E_{1\text{-}4}$	Δk_{12}	Δk_{13}	Δk_{14}	$FWHM_0$[1]	$k_{broadening}$[1]
**1** φ_n	**1**	< 1%	20 %	20 %	-	< 1%	< 1%
**1** Φ_n	**1**	< 1%	2 %	10 %	15 %	< 1%	< 1%
**2** φ_n	**2**	< 1%	3 %	3 %	-	< 1%	< 1%
**2** Φ_n	**2**	< 1%	2 %	2 %	5 %	< 1%	< 1%

[1] $FWHM_0$ refers to the theoretical FWHM at 0K and $k_{broadening}$ to the proportionality constant of the Doppler broadening (see equation S6.4)

As discussed above in the analysis of the lifetime fits, we explore the posterior parameter distribution using MCMC. The corner plot representation of the analysis is shown in Figure S5.6-9. We find strong negative correlation between the initial FWHM and the FWHM broadening factor which is expected. These values are of limited interest, and they do not strongly affect the estimation of the other parameters. For some fits, an increased correlation between Energy positions E_n and the corresponding rates can be found, also reflected in the uncertainty estimates in Table S5.4. Especially, a strong correlation between the rates of the second and third sublevel in the φ_n model of **1** can be found. Based on these results, the parameter estimates of the emissive rates and FWHM broadening should be handled with caution while the relaxation Energies of each sublevel are more stable.

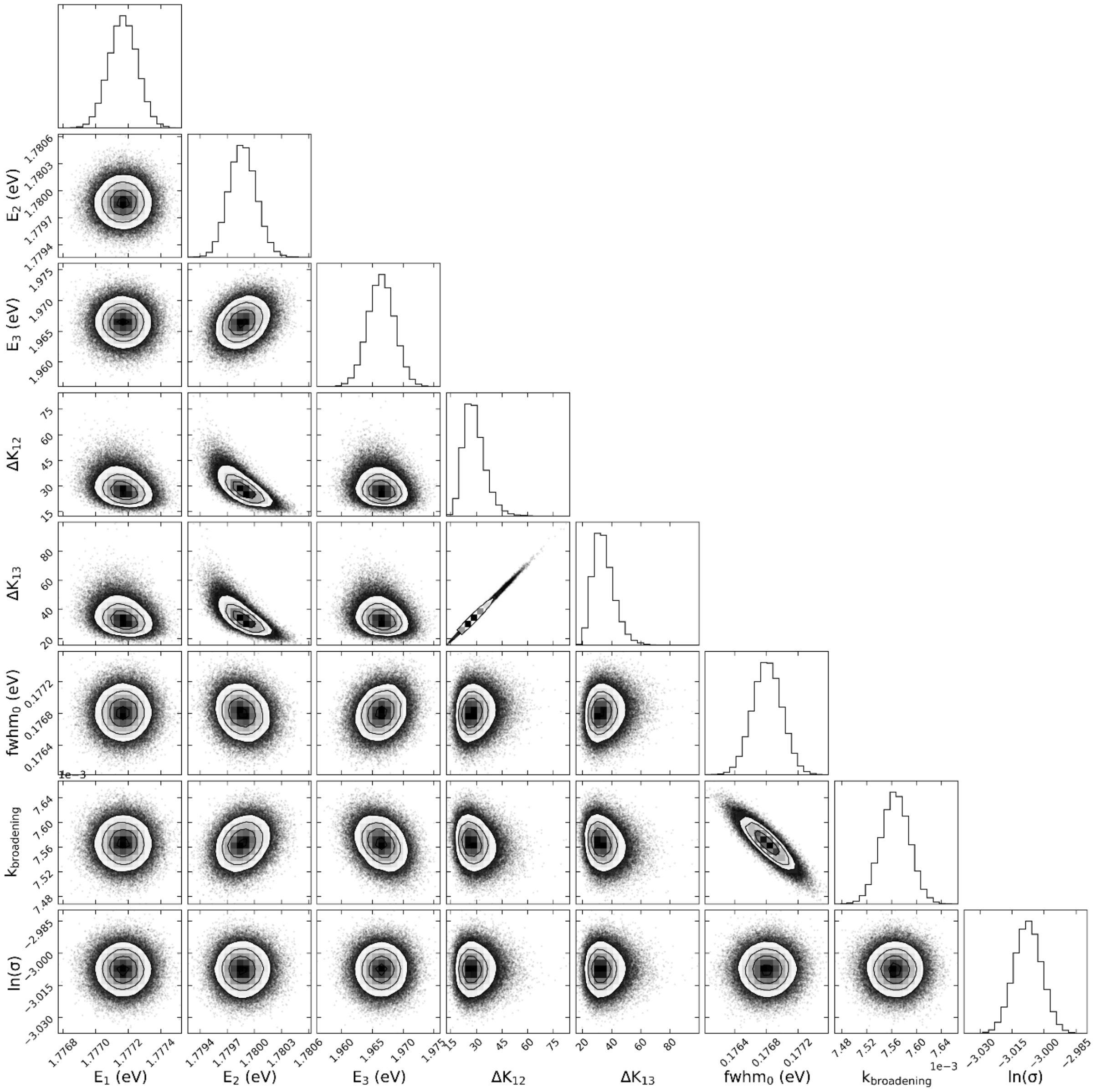

Figure S5.6 Corner Plots of the MCMC posterior parameter distribution of the spectral emission fits of **1** using the φ_n model. On the diagonal axis the marginalized distribution (uncertainty) of each parameter including the noise σ is shown in a histogram while the other plots show the correlation of two parameter sets.

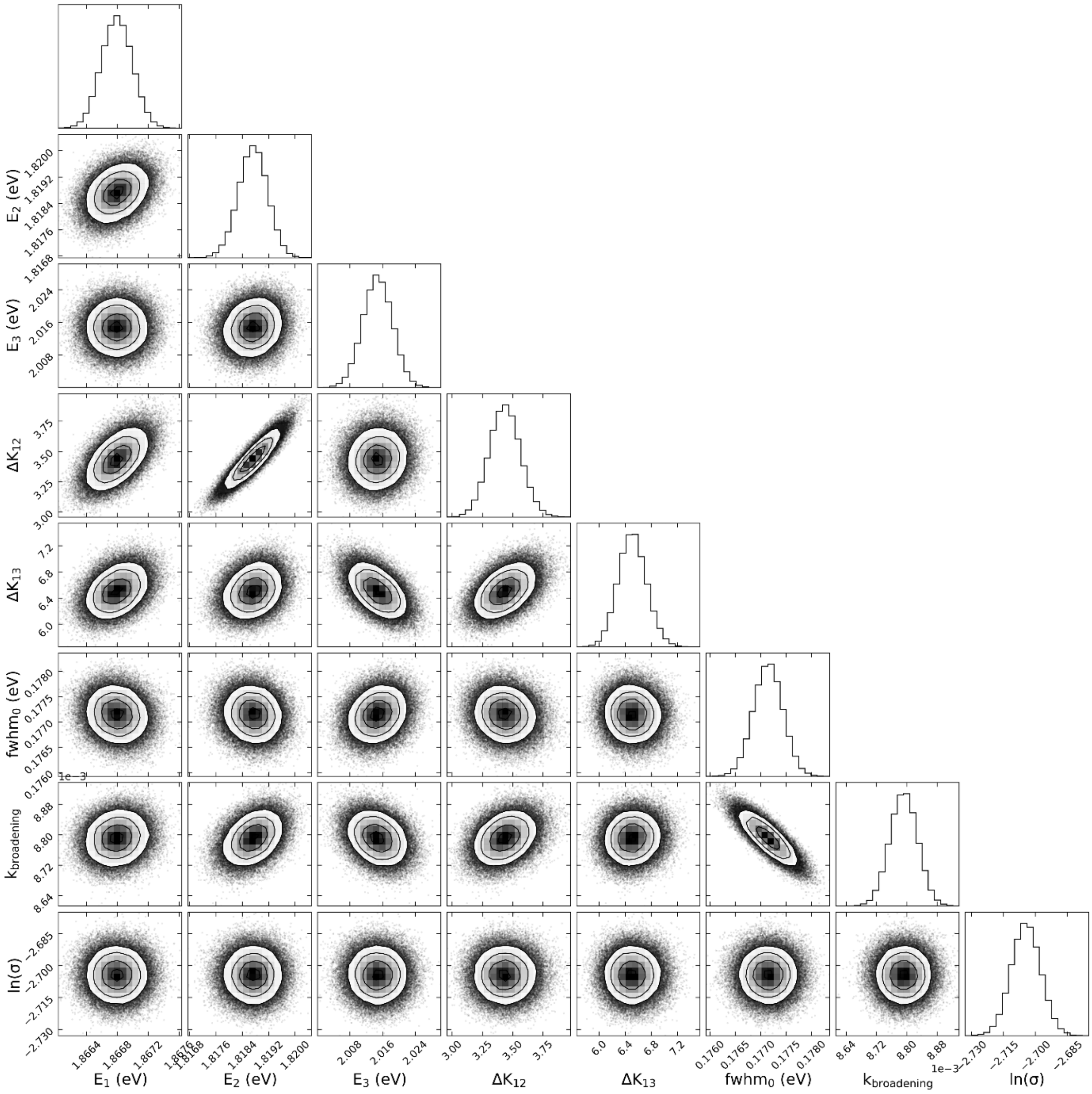

Figure S5.7 Corner Plots of the MCMC posterior parameter distribution of the spectral emission fits of **2** using the φ_n model. On the diagonal axis the marginalized distribution (uncertainty) of each parameter including the noise σ is shown in a histogram while the other plots show the correlation of two parameter sets.

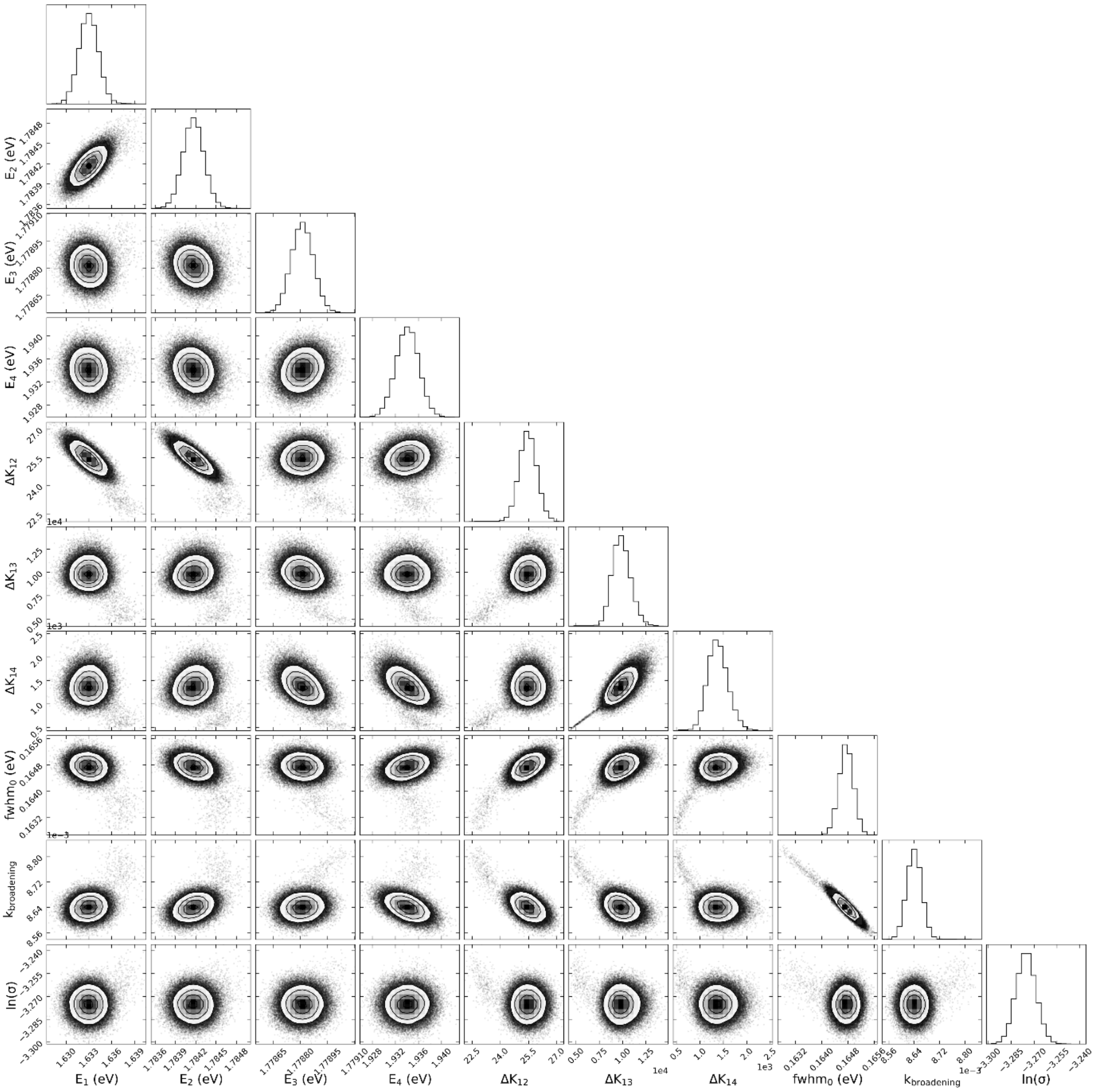

Figure S5.8 Corner Plots of the MCMC posterior parameter distribution of the spectral emission fits of **1** using the ϕ_n model. On the diagonal axis the marginalized distribution (uncertainty) of each parameter including the noise σ is shown in a histogram while the other plots show the correlation of two parameter sets.

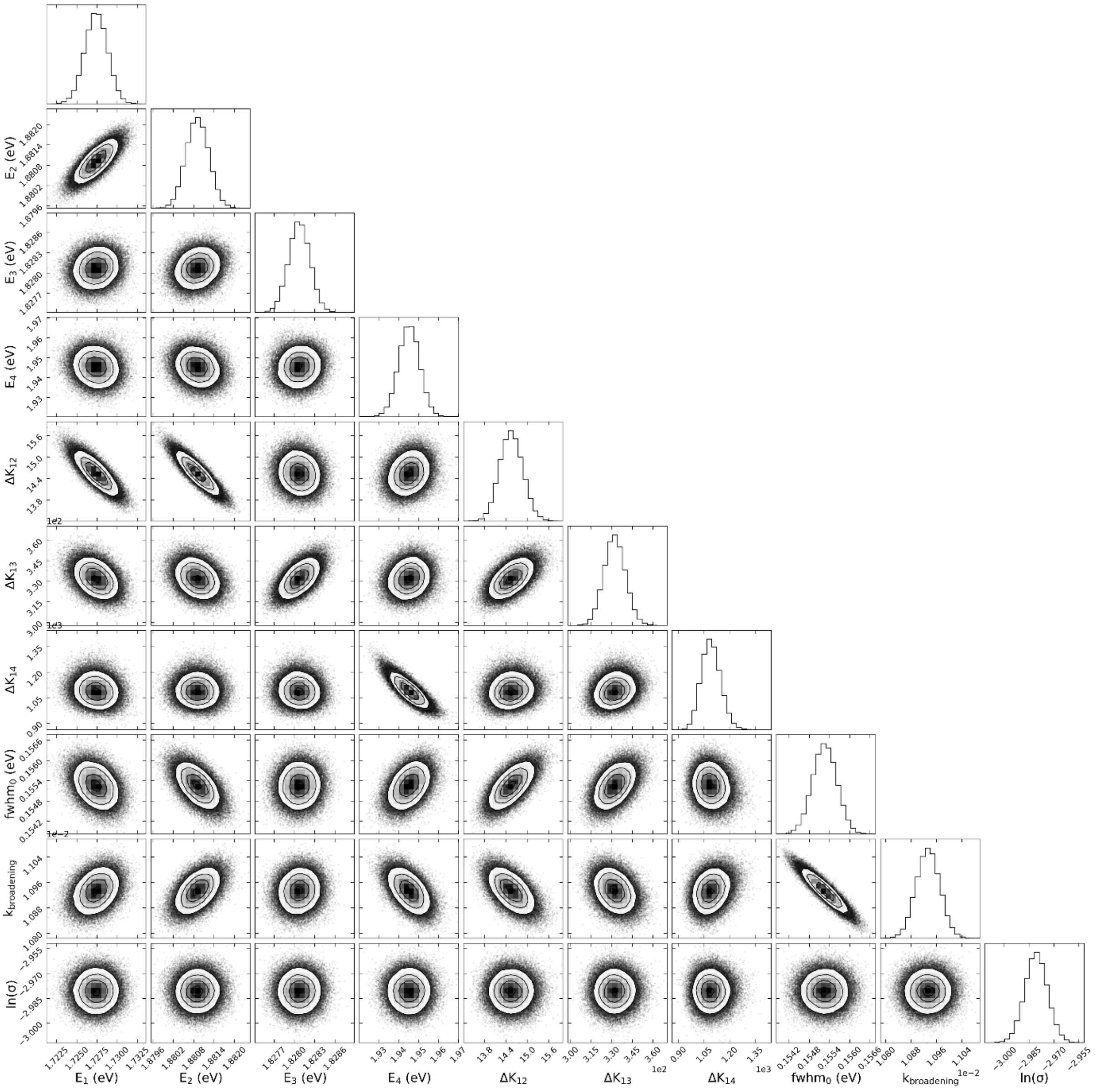

Figure S5.9 Corner Plots of the MCMC posterior parameter distribution of the spectral emission fits of **2** using the ϕ_n model. On the diagonal axis the marginalized distribution (uncertainty) of each parameter including the noise σ is shown in a histogram while the other plots show the correlation of two parameter sets.

S6 Additional Information on the φ_n/Φ_n model

Model Assumptions and Comparison:

The models irrespective of the number of sublevels assume that at every temperature, every emissive sublevel is in a Boltzmann equilibrium. Additionally, the spectral shape of the phosphorescence of every sublevel is similar and constant at a given temperature and may only vary in their peak position E_n. In its initial implementation by Azumi and colleagues, it was further assumed that the emission spectrum is independent of the temperature, meaning that the FWHM is constant over the whole temperature range.[6] As mentioned by Kitamura and coworkers, this is not warranted and temperature dependent spectral broadening should be considered.[7] Therefore, in their model implementation, they kept the FWHM constant for every sublevel at a given temperature but allowed a variation with temperature. However, they did not mention restrictions or a temperature dependence of the broadening. In our implementation of the φ_n/ϕ_n model, we assumed a homogeneous Doppler broadening leading to a monotonous increase in FWHM proportional to $\sqrt{T}$.

The degrees of degeneracy used for equation (2) in the main manuscript are based on the assumed geometries by the φ_n/Φ_n models. They are summarized in Table S6.1.

Table S6.1. Degree of degeneracy for the sublevels according to the geometry assumed by the φ_n/Φ_n model

**φ_n Model** Degree of degeneracy g_n			**Φ_n model** Degree of degeneracy g_n			
n = 1	n = 2	n= 3	n = 1	n = 2	n = 3	n= 4
3 (T_{2u})	2 (E_u)	3 (T_{1u})	2 (E_u)	1 (B_u)	2 (E_u)	3 (T_{1u})

Derivation of the spectral fit

Based on the calculated zero-magnetic-field splitting of the sublevels and the φ_n/Φ_n model, the emission spectra of Figure 4A can be simulated with the following equation

$$I(E,T) = \sum_n k_n * G(E - E_n) * P(T,\ \Delta E_{1n})\,, \qquad (S6.1)$$

where each sublevel n contributes with the product of the emissive rate constant k_n relative to φ_1 or Φ_1, the normalized gaussian function G(E-E_n) and the probability of overcoming the energy barrier ΔE_{1n} due to thermal energy P(T). G(E-E_n) and P(T) are defined as follows:

$$G(E - E_n, n) = \frac{1}{FWHM(\Phi_n)\sqrt{\frac{\pi}{2}}}\ e^{-2\frac{(E-E_n)^2}{FWHM^2}} \qquad (S6.2)$$

$$P(T,n) = e^{-\frac{\Delta E_{1n}}{k_B T}} \quad , (S6.3)$$

with E and T being the spectral energy and temperature. E_n and FWHM(Φ_n) describe the emission maximum energy and full width at half maximum of φ_n /Φ_n respectively. ΔE_{1n} refers to the energy difference between φ_1 or Φ_1 and φ_n or Φ_n ($e.g\ \Delta E_{11} = 0$) similar to equation 2 in the main manuscript. The FWHM for every sublevel is kept constant at a given temperature and a homogenous Doppler broadening $\left(\propto \sqrt{T}\right)$ is assumed, leading to the following model of the FWHM:

$$FWHM = FWHM_0 + k_{broadening} * \sqrt{T}\ , \quad (S6.4)$$

S7 TDDFT Analysis

Optimized Geometries for the SOC2 and SOC3 states of **1**

Chart S7.1. Distortions found in the SOC2 (A) and SOC3 (B) geometry (W = purple, I = green) compared to SOC0 geometry (W = red, I = yellow)

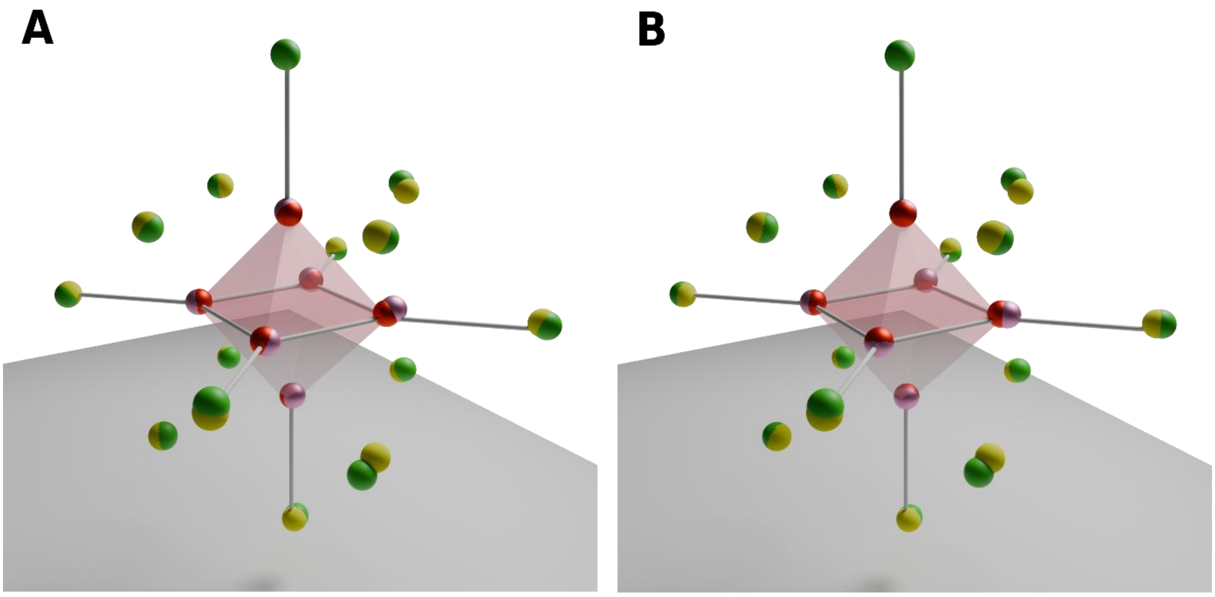

Eigenvectors of the SOC0-3 matrices of **1**

Table S7.1. Eigenvectors of the SOC0 matrices for 1

SOC0	Energy (eV)	ΔE_{1n} (meV)	State Mixing[1]			
			R	S	M_S	%
0	0	-	0	0	0	94 %
1	2.86	0	1	1	0	47 %
			1	1	-1	47 %
			1	1	1	5 %
2	2.86	0.2	1	1	1	42 %
			1	1	-1	42 %
			1	0	0	15 %
3	2.86	0.4	1	1	0	80 %
			1	1	-1	10 %
			1	1	1	10 %
4	2.90	38	3	1	0	39 %
			2	1	1	15 %
			2	1	-1	15 %
			2	1	0	10 %

[1] R represents the root or index of the non-relativistic state, S denotes the spin and Ms corresponds to the spin magnetic number

Table S7.2. Eigenvectors of the SOC1 matrices for 1

SOC1	Energy (eV)	ΔE_{1n} (meV)	State Mixing[1]			
			R	S	M_S	%
0	0	-	0	0	0	94 %
1	1.99	0	1	1	0	64 %
			1	1	1	16 %
			1	1	-1	16 %
2	2.00	4	1	1	1	48 %
			1	1	-1	48 %
			2	0	0	2 %
3	2.01	16	1	1	1	32 %
			1	1	-1	32 %
			1	1	0	30 %
4	2.34	350	1	0	0	84%
			2	1	1	4 %
			2	1	-1	4 %
			3	1	0	3 %

[1] R represents the root or index of the non-relativistic state, S denotes the spin and Ms corresponds to the spin magnetic number

Table S7.3. Eigenvectors of the SOC2 matrix of **1**

SOC2	Energy (eV)	ΔE_{1n} (meV)	State Mixing[1]			
			R	S	M_S	%
0	0	-	0	0	0	94 %
1	1.98	0	1	1	0	36 %
			1	1	1	30%
			1	1	-1	30 %
2	1.98	4	1	1	1	48 %
			1	1	-1	48 %
			1	1	0	1 %
3	1.99	14	1	1	0	58 %
			1	1	-1	19 %
			1	1	1	19 %
4	2.32	347	1	0	0	86%
			4	1	1	2 %
			4	1	-1	2 %
			3	1	-1	2 %

[1] R represents the root or state index, S denotes the spin and Ms corresponds to the spin magnetic number

Table S7.4. Eigenvectors of the SOC3 matrix of **1**

SOC3	Energy (eV)	ΔE_{1n} (meV)	State Mixing[1]			
			R	S	M_S	%
0	0	-	0	0	0	94 %
1	2.00	0	1	1	0	64 %
			1	1	1	16 %
			1	1	-1	16 %
2	2.00	4	1	1	1	48 %
			1	1	-1	48 %
			2	0	0	2 %
3	2.02	17	1	1	1	32 %
			1	1	-1	32 %
			1	1	0	30 %
4	2.35	349	1	0	0	84%
			2	1	1	4 %
			2	1	-1	4 %
			3	1	0	3 %

[1] R represents the root or index of the non-relativistic state, S denotes the spin and Ms corresponds to the spin magnetic number

Table S7.5. Cartesian coordinates in Å of the optimized SOC0 geometry of **1**.

I	10.51409878302265	15.03847191756634	0.20347215420503
I	10.12102030888199	15.72163732445811	4.03836786959419
I	7.42518882839472	13.56627602624270	2.10682590902797
I	9.64682752085683	18.65374628557627	1.42637117379342
I	6.95029126355422	16.49975576133775	-0.50378650106645
I	6.54443941312038	20.38568655447897	-0.21446926213751
I	3.89436113455103	14.31160799344930	0.59300176901459
I	7.67278943436324	13.38331413275191	6.00675996831038
I	10.32286314852287	19.45733600960282	5.19930388700604
I	4.09618936426873	18.04731646015082	1.75394844815905
I	4.57038983560981	15.11522275012976	4.36593817886835
I	7.26692645527650	17.26921484447534	6.29608943552474
I	6.79203384644629	20.20269412266689	3.68548588976401
I	3.70311862892982	18.73053309986188	5.58886078456506
W	8.45854515954427	16.15213162137497	1.82792755938521
W	6.88423643389314	18.27125509617078	1.66145510835013
W	5.83350767679465	15.86534758524160	1.98307706456901
W	8.38370974379449	17.90360992949121	3.80922976458479
W	7.33298247202941	15.49771120546363	4.13085176656154
W	5.75868054814480	17.61683127950862	3.96438903192037

Table S7.6. Cartesian coordinates in Å of the optimized SOC1 geometry of **1**.

I	10.52738571216460	15.03239448699424	0.19596483000912
I	10.12636263723803	15.71876019465783	4.02794051447128
I	7.41907068390729	13.57544490923704	2.11372300086308
I	9.63765341750521	18.65192873860963	1.43300572202866
I	6.96280120508602	16.49421233864647	-0.50703267362456
I	6.59509949593212	20.43390557502452	-0.16470320253365
I	3.89522268213675	14.25877794042944	0.65601854982862
I	7.60447368798389	13.34663789357841	5.97288499569907
I	10.30136831976004	19.52405804948257	5.14629255456436
I	4.06378786867142	18.02220649499691	1.44190591213995
I	4.54750679722975	15.17904158704813	4.39069847656751
I	7.54607792801706	17.16590923383515	6.42210170423598
I	6.73142560382292	20.18136639692413	3.72316630568370
I	3.64832222674874	18.75488176432787	5.63400847355186
W	8.46977559918278	16.14574065151214	1.81926756208626
W	6.87484700819918	18.26811748671446	1.65156188072237
W	5.82774382695162	15.87201764360003	1.97150303397368
W	8.38702881987432	17.89724729502251	3.82026583687782
W	7.34066977640725	15.50192845572710	4.14100916002821
W	5.66557670318063	17.66512286363069	4.03351736282649

Table S7.7. Cartesian coordinates in Å of the optimized SOC2 geometry of **1**.

I	10.50897902495057	15.04008846342761	0.16748395085157
I	10.14492102633883	15.70762046346178	4.01026823585061
I	7.41711174881893	13.57594501848987	2.10583320485055
I	9.63427372788446	18.65409613134115	1.42778195983372
I	6.94219200359880	16.50475057087487	-0.49348625399788
I	6.60441440910880	20.43942391134083	-0.14959775072309
I	3.89988777800718	14.24719440004639	0.67722078617179
I	7.62908483879891	13.35289951279238	5.98714237472682
I	10.31492405809041	19.49930927382655	5.17106154924143
I	4.04975940601002	18.01513902936544	1.37277695642215
I	4.55309107310486	15.17901095413045	4.39432770878174
I	7.45711269893598	17.19714948245275	6.38770550839196
I	6.73969174202670	20.18063825207384	3.72393803824824
I	3.62493620205682	18.76721177190653	5.59226221413980
W	8.46782638218377	16.14689798613635	1.81614452944396
W	6.86411529717155	18.26868362734729	1.67137554341840
W	5.82273557244438	15.88335884968404	1.98991965010687
W	8.41121588679456	17.89341251212783	3.81916300220026
W	7.35975442126326	15.48555273368033	4.14044826589033
W	5.72617270241077	17.65131705549272	4.11133052615056

Table S7.8. Cartesian coordinates in Å of the optimized SOC3 geometry of **1**.

I	10.52679561045318	15.03358605078175	0.19589510543466
I	10.12650620072251	15.71890135393289	4.02782498549781
I	7.41907033837782	13.57553461133979	2.11333903783289
I	9.63723246498221	18.65278738437124	1.43378488676591
I	6.96241200287767	16.49428837183643	-0.50680417261076
I	6.59416089267519	20.43271646724594	-0.16684565027807
I	3.89528098301892	14.25839477667693	0.65611882628100
I	7.60629549343272	13.34545701767922	5.97256642004991
I	10.30180928628762	19.52207689877469	5.14857736818093
I	4.06290568171603	18.02211638470659	1.44371118443695
I	4.54831910151454	15.17846231674968	4.39114978087431
I	7.54239568137980	17.16564816206722	6.42195841854580
I	6.73346886589011	20.18228886998781	3.72154247237317
I	3.64969274907505	18.75648452090796	5.63225891208607
W	8.46956001748266	16.14625651671996	1.81950680134102
W	6.87430350036185	18.26853684952030	1.65116701376172
W	5.82735171804911	15.87220257775240	1.97163225749458
W	8.38698235381535	17.89727103999266	3.82117328567431
W	7.34107243039201	15.50108908401826	4.14165775517855
W	5.66658462749533	17.66560074493761	4.03288531107910

Table S7.9. Structural parameters in Å of the optimized SOC0, SOC1, SOC2 and SOC3 geometries of **1**.

Distances in Å	SOC0	SOC1	SOC2	SOC3
W-W average	2.6452	2.6837	2.6841	2.6839
W-W min	2.6450	2.6335	2.6222	2.6341
W-W max	2.6455	2.7396	2.7632	2.7388
W-I^{inner} average	2.7988	2.8254	2.8042	2.8253
W-I^{inner} min	2.7984	2.7492	2.7511	2.7504
W-I^{inner} max	2.7993	3.0807	2.8956	3.0784
W-I^{apical} average	2.8470	2.8343	2.8348	2.8341
W- I^{apical} min	2.8468	2.7962	2.8024	2.7957
W- I^{apical} max	2.8472	2.8475	2.8477	2.8472

Optimized Geometries for the S_0 and T_1 states of **2**

Chart S7.2. Distortions found in the T_1 geometry (W = purple, I = green, C = light gray, O = pink) compared to S_0 geometry (W = red, I = yellow, C = dark gray, O = orange). Fluorine atoms are omitted for clarity.

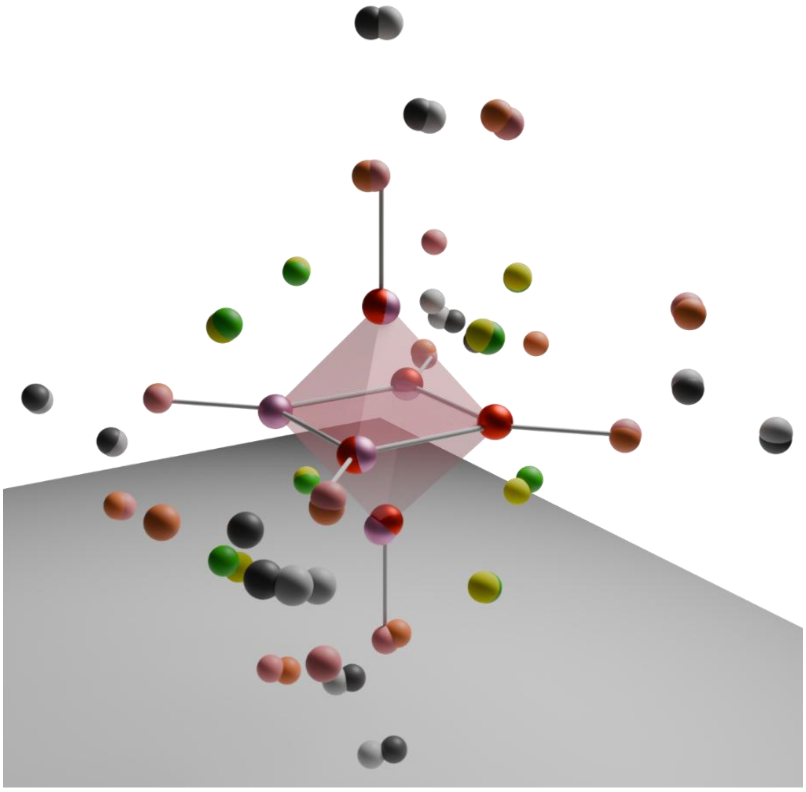

Table S7.10. Cartesian coordinates in Å of the optimized S_0 geometry of **2**.

W	1.43535822261459	0.34368398027799	1.16731691661541
W	-0.01980848108324	-1.80435013857941	0.55600365561818
W	-1.21134735691340	0.43127372730314	1.36208970771758
I	2.79287651654736	-2.01382519865113	0.38154066834231
I	0.21606526274075	-1.07633055238847	3.24978980380333
I	0.25455766433193	2.71508769526192	2.09753407948120
I	2.81879061092566	1.82717080869732	-0.79432802313493
O	3.13637620496732	0.69168888926466	2.36801484885251
C	3.31536901269670	1.06067455902001	3.57659666976323
O	2.51698085521143	1.34350473004459	4.43782271105440
C	4.83320003662091	1.12812008170849	3.89817972600455
F	5.46411508156469	1.99726202894874	3.09482835155112
F	5.42079989362914	-0.06600471036528	3.73177446376704
F	5.06606261966084	1.51083149268614	5.15362758685136
O	0.03675516427401	-3.85052207681359	1.09399560312678
C	-0.52917150871450	-4.53764760506169	2.00660356038509
O	-1.31399196532418	-4.20956861092149	2.86647296632497
C	-0.07919250952608	-6.02160883054888	1.92196403564589
F	-0.64884889880009	-6.77570730480577	2.86241533677687
F	-0.39597273234195	-6.56036049863146	0.73557452040699
F	1.24844245872655	-6.13778531101321	2.07041005991159
O	-2.68419755170348	0.89737433139014	2.79549078756252
C	-2.65433123148445	1.26964175161517	4.01658703201633
O	-1.71782042153757	1.46660874431881	4.75322854049113
C	-4.09718483093659	1.48629262754734	4.54784427389841

F	-4.72502329460631	2.45265185934631	3.85996981484624
F	-4.11248443324853	1.84317764296706	5.83200810161958
F	-4.83558301785910	0.37399199417018	4.43509027297521
W	-1.43536221413462	-0.34368357305982	-1.16731307764444
W	0.01980391450641	1.80435128593728	-0.55599943286558
W	1.21134349684642	-0.43127307366725	-1.36208562463083
I	-2.79288078209530	2.01382441071866	-0.38153350434539
I	-0.21606872925268	1.07633190766972	-3.24978494949548
I	-0.25456288329436	-2.71508711376782	-2.09752903820659
I	-2.81879602134063	-1.82716853838420	0.79433118710521
O	-3.13637363832371	-0.69168900630156	-2.36801639737692
C	-3.31535730876878	-1.06067535402855	-3.57659879995543
O	-2.51696339564787	-1.34349616170035	-4.43782230736204
C	-4.83318610661349	-1.12813255851245	-3.89818927903420
F	-5.46410177247272	-1.99727126085756	-3.09483492695811
F	-5.42079236294494	0.06599087117264	-3.73179606311685
F	-5.06604044026736	-1.51085438111281	-5.15363520165576
O	-0.03675732499961	3.85052317967194	-1.09398951429001
C	0.52916803658785	4.53764536761797	-2.00660062640540
O	1.31398469617036	4.20956293358228	-2.86647171829192
C	0.07918885103210	6.02160673115613	-1.92196537726855
F	0.64886770462118	6.77570947194166	-2.86239930313339
F	0.39593731172377	6.56035539697069	-0.73556637106980
F	-1.24844303795877	6.13778092537809	-2.07044513941470
O	2.68419591193027	-0.89737217116947	-2.79548599929444
C	2.65432775908005	-1.26964222928880	-4.01658135918194
O	1.71781579337198	-1.46661604916623	-4.75321902708458
C	4.09718114312602	-1.48628030549730	-4.54784348855036
F	4.72504599913847	-2.45260690426472	-3.85994790598648
F	4.11247845768917	-1.84319987693186	-5.83199771352421
F	4.83555557185840	-0.37396003089400	-4.43512511323670

Table S7.11. Cartesian coordinates in Å of the optimized T_1 geometry of **2**.

W	1.40944827849183	0.32992321336323	1.16991488173629
W	-0.03065856292294	-1.79937263827109	0.54968338943503
W	-1.24937210496657	0.41414223344245	1.48952891668598
I	2.77857587698001	-2.00980260860493	0.37110568900087
I	0.42058618573276	-1.28090773431972	3.29372553295073
I	0.15837196479311	2.73191836733587	2.07491253245906
I	2.80612457262711	1.81723575031530	-0.76646019697292
O	3.07309733386996	0.74575982707131	2.40388229825260
C	3.20157312810654	1.19401521049833	3.58969433049784
O	2.35744405883513	1.47355945325566	4.41318443357327
C	4.69968568351920	1.39018930395362	3.93755043849218
F	5.26812450375408	2.26837162939087	3.10100659574693
F	5.37437640374640	0.23896248287258	3.83044711969953
F	4.86965838910280	1.84476118241556	5.17580756921484
O	-0.03618923290362	-3.85251654884165	1.06360785909176
C	-0.67225213758653	-4.53242453299321	1.93296395342609
O	-1.47748237570756	-4.17452133457761	2.76524115050471
C	-0.30811384884682	-6.03537790315265	1.82597796194895
F	-0.92486961173269	-6.76531281056191	2.75105135839633
F	-0.65606159904928	-6.52074117283727	0.62667380640237
F	1.00876603850979	-6.22529975712305	1.96989313273982
O	-2.66031910517404	0.86334238534564	2.96393807210867
C	-2.54880337895292	1.22553122443929	4.18428579736588
O	-1.55163109606000	1.41322302613632	4.84427849245410
C	-3.94346507874395	1.43499709980726	4.82694752444660
F	-4.61417438480755	2.40484136376107	4.19267021072170
F	-3.85525064371469	1.77695308505147	6.10881066978462
F	-4.68013664040781	0.32031093077988	4.75285968461140
W	-1.42421137900590	-0.32010435282223	-1.17898659529481

W	-0.00207002709109	1.78443061065199	-0.57133051013366
W	1.21003509024400	-0.43711227196654	-1.36379814750861
I	-2.84163569079120	2.13284219649638	-0.74985576786413
I	-0.18606109898257	1.07732701529276	-3.25625609585307
I	-0.26476216098524	-2.72227860518761	-2.08376589781438
I	-2.87252182491969	-1.74612410117811	0.78830882827792
O	-3.06511887005791	-0.74882774162335	-2.43279394959816
C	-3.19710103939893	-1.11699821837816	-3.64532436227131
O	-2.35800917841641	-1.29574174635101	-4.50112150122464
C	-4.69301918666075	-1.34417725402536	-3.98390183017880
F	-5.23841148389395	-2.24723748093957	-3.15994336577616
F	-5.38875280827055	-0.20758080039748	-3.85145190886032
F	-4.86270284356491	-1.77913290697439	-5.22930596339079
O	0.02466951133186	3.82675969380464	-1.11430659855406
C	0.61372143224402	4.50150535541873	-2.01989495786201
O	1.36101399929919	4.13717249685879	-2.90187657250782
C	0.26501426637623	6.00683344840217	-1.89325537446020
F	0.86382225871797	6.73797126415555	-2.82915083674671
F	0.64332877066159	6.48391130078803	-0.70056054128246
F	-1.05425526167185	6.20453506147090	-2.00566352248227
O	2.66656219594165	-0.90418429555806	-2.82797851386919
C	2.59166038716292	-1.26221443824750	-4.04878291667262
O	1.61875248870865	-1.46133273145444	-4.74341309768264
C	4.00790218732006	-1.45324480667047	-4.65077191860936
F	4.67267478025991	-2.41714186813503	-4.00049637092263
F	3.96354769554469	-1.79175531435517	-5.93617233110333
F	4.72887517340651	-0.32986023702813	-4.55133258452905

Table S7.12. Structural parameters in Å of the optimized S_0 and T_1 geometries of **2**.

Distances in Å	S_0	T_1
W-W average	2.6602	2.6769
W-W min	2.6549	2.6117
W-W max	2.6659	2.7732
W-I^{inner} average	2.8163	2.8238
W-I^{inner} min	2.8000	2.7743
W-I^{inner} max	2.8365	2.9861

References

(1) Sokolov, M. N.; Brylev, K. A.; Abramov, P. A.; Gallyamov, M. R.; Novozhilov, I. N.; Kitamura, N.; Mikhaylov, M. A. Complexes of $\{W_6I_8\}^{4+}$ Clusters with Carboxylates: Preparation, Electrochemistry, and Luminescence. *Eur. J. Inorg. Chem.* **2017**, *2017* (35), 4131–4137. https://doi.org/10.1002/ejic.201700618.

(2) Riehl, L.; Seyboldt, A.; Ströbele, M.; Enseling, D.; Jüstel, T.; Westberg, M.; Ogilby, P. R.; Meyer, H.-J. A Ligand Substituted Tungsten Iodide Cluster: Luminescence vs. Singlet Oxygen Production. *Dalton Trans.* **2016**, *45* (39), 15500–15506. https://doi.org/10.1039/C6DT02471H.

(3) Pachel, F.; Frech, P.; Ströbele, M.; Enseling, D.; Romao, C. P.; Jüstel, T.; Scheele, M.; Meyer, H.-J. Preparation, Photoluminescence and Excited State Properties of the Homoleptic Cluster Cation $[(W_6I_8)(CH_3CN)_6]^{4+}$. *Dalton Trans.* **2023**, *52* (12), 3777–3785. https://doi.org/10.1039/D2DT04063H.

(4) Foreman-Mackey, D.; Hogg, D. W.; Lang, D.; Goodman, J. Emcee: The MCMC Hammer. *Publ. Astron. Soc. Pac.* **2013**, *125* (925), 306. https://doi.org/10.1086/670067.

(5) Foreman-Mackey, D. Corner.Py: Scatterplot Matrices in Python. *J. Open Source Softw.* **2016**, *1* (2), 24. https://doi.org/10.21105/joss.00024.

(6) Miki, H.; Ikeyama, T.; Sasaki, Y.; Azumi, T. Phosphorescence from the Triplet Spin Sublevels of a Hexanuclear Molybdenum(II) Chloride Cluster Ion, $[Mo_6Cl_{14}]^{2-}$: Relative Radiative Rate Constants for Emitting Sublevels. *J. Phys. Chem.* **1992**, *96* (8), 3236–3239. https://doi.org/10.1021/j100187a012.

(7) Kitamura, N.; Kuwahara, Y.; Ueda, Y.; Ito, Y.; Ishizaka, S.; Sasaki, Y.; Tsuge, K.; Akagi, S. Excited Triplet States of $[\{Mo_6Cl_8\}Cl_6]^{2-}$, $[\{Re_6S_8\}Cl_6]^{4-}$, and $[\{W_6Cl_8\}Cl_6]^{2-}$ Clusters. *Bull. Chem. Soc. Jpn.* **2017**, *90* (10), 1164–1173. https://doi.org/10.1246/bcsj.20170168.
